# What is the contribution of voluntary and reflex processes to sensorimotor control of balance?

**DOI:** 10.3389/fbioe.2022.973716

**Published:** 2022-09-29

**Authors:** Amel Cherif, Jacopo Zenzeri, Ian Loram

**Affiliations:** ^1^ Department of Robotics, Brain and Cognitive Sciences, Istituto Italiano di Tecnologia, Genoa, Italy; ^2^ Cognitive Motor Function Research Group, Research Centre for Musculoskeletal Science & Sports Medicine, Dept of Life Sciences, Faculty of Science and Engineering, Manchester Metropolitan University, Manchester, United Kingdom

**Keywords:** balance, reflex, voluntary, postural control, intermittent control, refractoriness

## Abstract

The contribution to balance of spinal and transcortical processes including the long-latency reflex is well known. The control of balance has been modelled previously as a continuous, state feedback controller representing, long-latency reflexes. However, the contribution of slower, variable delay processes has not been quantified. Compared with fixed delay processes (spinal, transcortical), we hypothesize that variable delay processes provide the largest contribution to balance and are sensitive to historical context as well as current states. Twenty-two healthy participants used a myoelectric control signal from their leg muscles to maintain balance of their own body while strapped to an actuated, inverted pendulum. We study the myoelectric control signal (u) in relation to the independent disturbance (d) comprising paired, discrete perturbations of varying inter-stimulus-interval (ISI). We fit the closed loop response, u from d, using one linear and two non-linear non-parametric (many parameter) models. Model M1 (ARX) is a generalized, high-order linear-time-invariant (LTI) process with fixed delay. Model M1 is equivalent to any parametric, closed-loop, continuous, linear-time-invariant (LTI), state feedback model. Model M2, a single non-linear process (fixed delay, time-varying amplitude), adds an optimized response amplitude to each stimulus. Model M3, two non-linear processes (one fixed delay, one variable delay, each of time-varying amplitude), add a second process of optimized delay and optimized response amplitude to each stimulus. At short ISI, the myoelectric control signals deviated systematically both from the fixed delay LTI process (M1), and also from the fixed delay, time-varying amplitude process (M2) and not from the two-process model (M3). Analysis of M3 (all fixed delay and variable delay response amplitudes) showed the variable (compared with fixed) delay process 1) made the largest contribution to the response, 2) exhibited refractoriness (increased delay related to short ISI) and 3) was sensitive to stimulus history (stimulus direction 2 relative to stimulus 1). For this whole-body balance task and for these impulsive stimuli, non-linear processes at variable delay are central to control of balance. Compared with fixed delay processes (spinal, transcortical), variable delay processes provided the largest contribution to balance and were sensitive to historical context as well as current states.

## Introduction

Sensorimotor control, including regulation of balance, combines feedback from multiple reflex and voluntary neural processes (Brooks, 1986). Spinal, transcortical, and additional indirect central loops through the basal ganglia and cerebellum allow modulation of muscle activity in the lower limb at latencies of 50–80 ms (spinal), 90–120 ms, (transcortical) and up to 500 ms or more (variable delay central loops) (Brooks, 1986; Caligiore et al., 2017; Crevecoeur and Kurtzer, 2018). The most reflexive processes (spinal, transcortical) we call “fixed latency” because they are characterized by small variation in latency limited to tens of milliseconds (Brooks, 1986). The more voluntary processes we call “variable delay” because they are characterized by large variation in latency of hundreds of milliseconds (Brooks, 1986; Loram et al., 2014). The flow of information around central sub-cortical loops (e.g., cortex, basal ganglia/cerebellum, thalamus, cortex) allows variable time to resolve multiple complex inputs before selecting a motor response from the cortical sensory input (Cohen and Frank, 2009; Frank, 2011; Shine, 2021). We propose that “variable delay” processes provide a substantial contribution to real-time balance and represent a sequential process of threshold triggered responses with variable latency similar to sub-movements observed in manual control (Loram et al., 2014; Loram et al., 2015a).

The “fixed delay” balance responses of the lower limb are dominated by the fastest transcortical component at 90–120 ms, named as long-latency reflex (Safavynia and Ting, 2013a). These transcortical responses can be modulated in amplitude according to intention, the current state of the body, and by multimodal proprioceptive, vestibular, cutaneous and visual sensory input (Pruszynski and Scott, 2012). While the amplitude of this long-latency transcortical reflex can be modulated, online modulation of response direction (positive/negative) relative to stimulus direction requires processes of even longer latency (Brooks, 1986; Day and Lyon, 2000; Loram et al., 2011; Loram et al., 2014).

Until recently, the control of balance has been conceptualized and modelled most successfully as time delayed, continuous, linear-time-invariant (LTI), state feedback representing long-latency reflexes (van der Kooij and de Vlugt, 2007; Kiemel et al., 2011; van der Kooij and Peterka, 2011; Safavynia and Ting, 2013b). For both upper and lower limbs, the long-latency reflex includes spinal and transcortical components summing linearly, and represents a feedback control process achieving task level goals rather than simple triggered reactions (Pruszynski et al., 2011). Referencing continuous linear reconstructions of muscle EMG signals from whole body center of mass (CoM) position, velocity and acceleration during perturbations to balance, using a best fit delay compatible with the long-latency reflex (Safavynia and Ting, 2013a; Safavynia and Ting, 2013b), it has been argued that long-latency reflexes reflect a continuous state feedback controller rather than an intermittent or direct controller (Crevecoeur and Kurtzer, 2018). For the upper limb, evidence from paired perturbations at inter-stimulus-intervals (ISI) of 35, 60 and 110 ms, ruled out refractoriness (delays related to ISI) at these ISI and supported the idea that long-latency reflexes implement continuous action of controllers with fixed function (Kurtzer, 2019). However, a recent analysis using high quality disturbance-balance data, showed a standard, time delayed, continuous, linear-time-invariant (LTI), state-estimation, state feedback model structure with added noise could not replicate concurrently the linear response, the remnant and observed time delays (Loram et al., 2022). The remnant remaining after subtraction of the linear response comprises 70–80% of the control signal, so most of the control signal is not generated by linear processes (Loram et al., 2022). This previous data, which sets a current benchmark representing whole body balance control, required a state-predictor (108 ± 40 ms) to reproduce the observed time delays concurrently with the linear response and remnant (Loram et al., 2022). Furthermore, the most comprehensive fit was achieved by a non-linear intermittent, rather than linear continuous, predictive control model (Loram et al., 2022). These previous results support the hypothesis that balance is non-linear and involves processes beyond long-latency reflex control.

For the upper limb, the concept of sequential, intermittent predictive control has substantial support (Fishbach et al., 2007; Houk et al., 2007; van de Kamp et al., 2013; Loram et al., 2014; Zenzeri et al., 2014; Morgan et al., 2021). Previous studies have decomposed upper limb reaching movements and sustained manual control into sub-movements (Milner, 1992; Rohrer and Hogan, 2003; Fishbach et al., 2007; Goble and Brown, 2007; van de Kamp et al., 2013). Specifically visually guided manual tracking shows observable sub-movements and variable stimulus-response delays of up to 500 ms or more (van de Kamp et al., 2013). These variable delays have been associated with event triggered intermittent control (Loram et al., 2015a; Gollee et al., 2017), and with refractoriness related to sequential processes selecting responses from multiple possibilities (Dux et al., 2006; Levy et al., 2006; Loram et al., 2014). While variable delay regulation of upper limb visuomotor control is accepted (Pruszynski et al., 2008; Manning et al., 2012; Battaglia-Mayer et al., 2015), regulation of whole body human balance, as above, is interpreted most typically as a continuous linear process using a single delay defined by the fastest transcortical response times (van der Kooij and de Vlugt, 2007; Welch and Ting, 2009; Safavynia and Ting, 2013a; Safavynia and Ting, 2013b; Crevecoeur and Kurtzer, 2018).

The literature on human balance is incomplete because the contribution of variable delay processes (voluntary response) has not been quantified. This later portion of the balance response is very substantial (c.f. “plateau region” and beyond in Figure 6 of (Welch and Ting, 2009)). The latency of this later portion means it can receive contributions from variable delay cerebella-basal ganglia-thalamic loops (Cohen and Frank, 2009; Frank, 2011; Shine, 2021), which is relevant because these central structures are implicated in neurological disorders of balance including Parkinson’s and cerebella ataxia.

**FIGURE 1:**
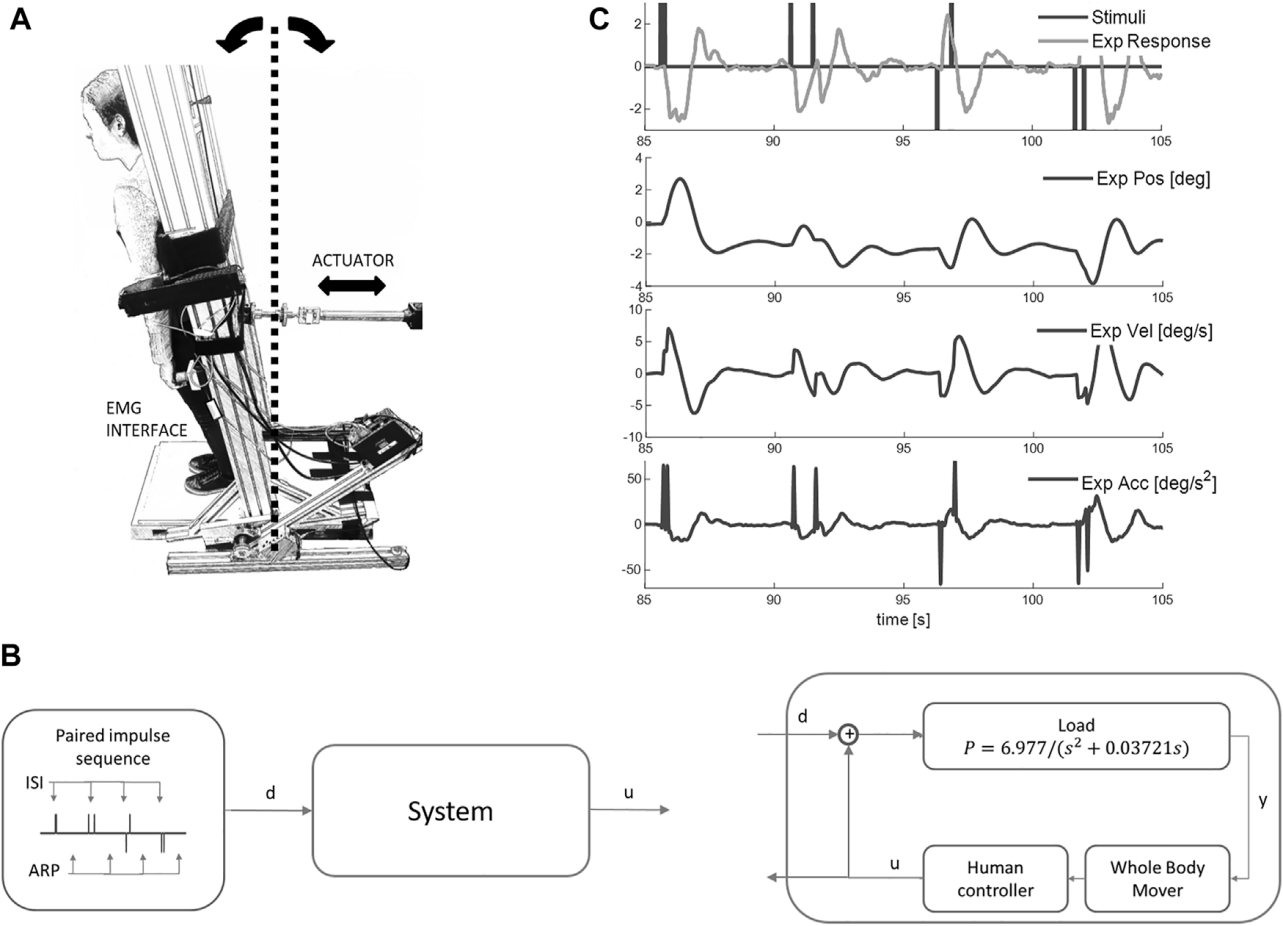
Balance task and response to impulse perturbations. **(A)** Participants, strapped to a one degree of freedom device with second order dynamics of upright standing, used visual-haptic-vestibular feedback and myoelectric control signals from the calf and tibialis anterior muscles to maintain balance for 250s. In this study the second order dynamics were set to be marginally stable: these setting ensured closed loop sway resembled natural postural sway most closely. **(B)** An input disturbance of discrete impulses (d) was applied and participants were asked to maintain balance (ISI: inter-stimuli interval between impulses; ARP: approximate recovery period; d: disturbance; y: load position; u: myoelectric control signal). **(C)** Representative disturbance (black upper), control signal (grey, upper), board angle, board velocity and board acceleration v time (s). Net myoelectric control signal responds to the discrete impulses.

Here we study whole body balance with the same task and apparatus reported previously to acquire data setting current benchmark quality (Loram et al., 2022). Participants use their natural senses and an integrated myoelectric control from their own leg muscles to control movement of their own body while strapped to an actuated, single segment robot (Figure 1). We use paired, discrete force perturbations d of variable ISI (0.15, 0.25, 0.35, 0.55, 0.85, 1.45, 2.45, 4.05 s), each producing a well-defined response u (Figure 1).

Our hypothesis is that balance is defined mainly by variable delay processes and that fixed delay processes (long-latency reflex) are a preliminary, incompletely formed part to the main response.

Typically, model-based hypothesis testing of the closed-loop balance control system (“System”, Figure 1) follows two stages. Stage 1 is a non-parametric (many parameter) analysis with minimal preconceptions to capture as fully as possible the control response u coherent with the perturbation d and its remnant. Stage 2 tests parametric (minimal parameter) control models to fit the non-parametric description of coherent perturbation response and remnant (Pintelon and Schoukens, 2001; van der Kooij and de Vlugt, 2007; Gollee et al., 2012; Gollee et al., 2017; Loram et al., 2022). In this study, we focus entirely on non-parametric analysis to capture as fully as possible the response u to perturbation d. In this study we treat the closed loop balance system as a “black box”. Some studies seek to identify processes within the system such as the feedback pathway between whole body mover position y and control signal u (Kiemel et al., 2011; Engelhart et al., 2015). However, to test our hypotheses there is no need to identify transfer functions within the black box system.

**FIGURE 2:**
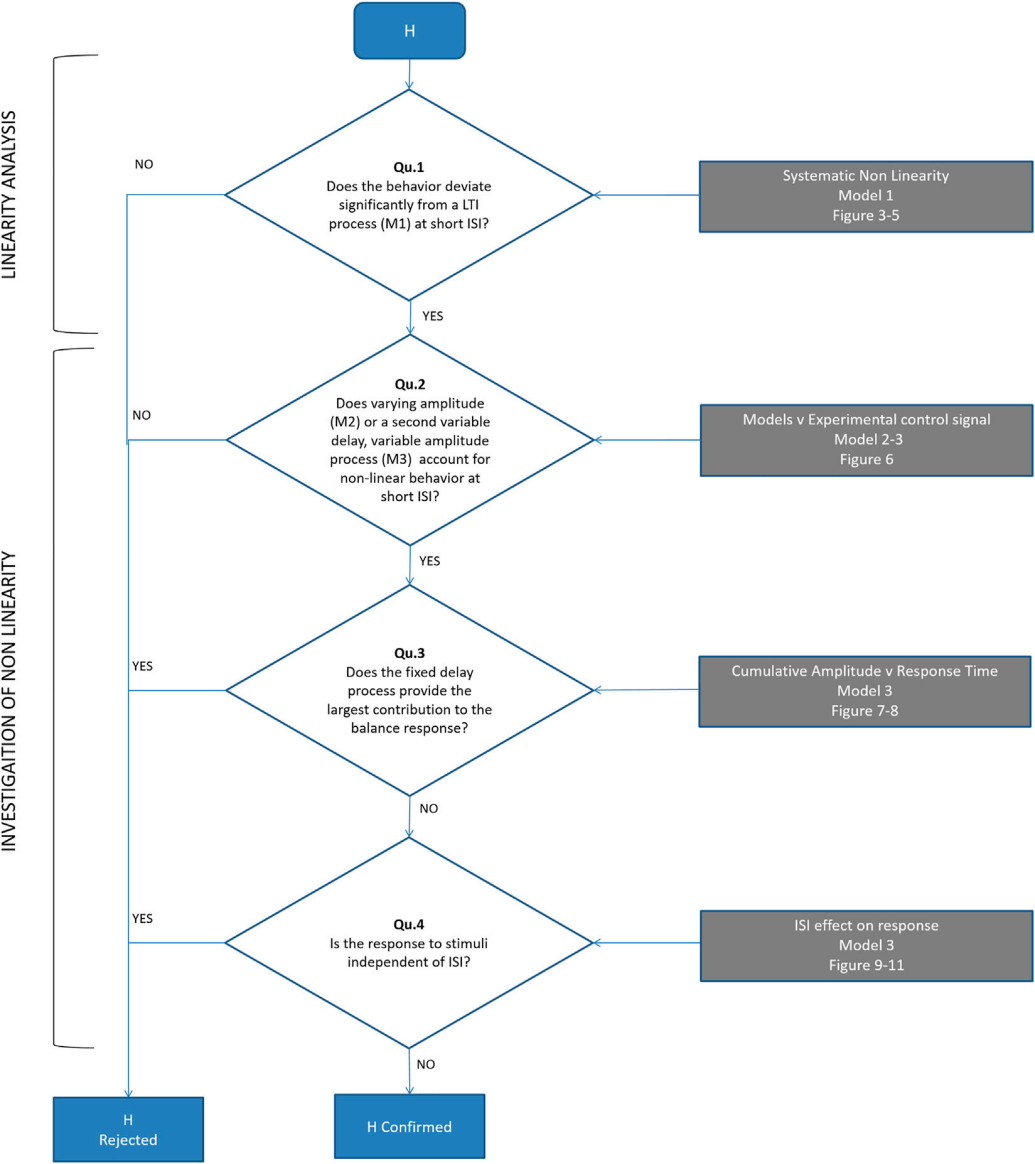
Flowchart to test our hypothesis (H): a single (reflexive) pathway provides the minor contribution to balance. Qu 1) Does the experimental control signal u deviate significantly from a LTI process (M1) at short ISI? (Figures 4, 5). Qu 2) Does time varying amplitude (M2) or a second variable delay, variable amplitude process (M3) account for non-linear behavior of the control signal u at short ISI? (Figure 6). Qu 3) Does the fixed delay process provide the largest contribution to the balance response? We calculated the cumulative amplitude v time of all discrete fixed and variable delay responses from model M3 (Figures 7, 8). Qu 4) Is the response to stimuli independent of ISI? We test the cumulative amplitude v time response for effect of ISI (Figures 9–11).

Our sequence of questions and hypothesis testing is presented in Figure 2.

**FIGURE 3:**
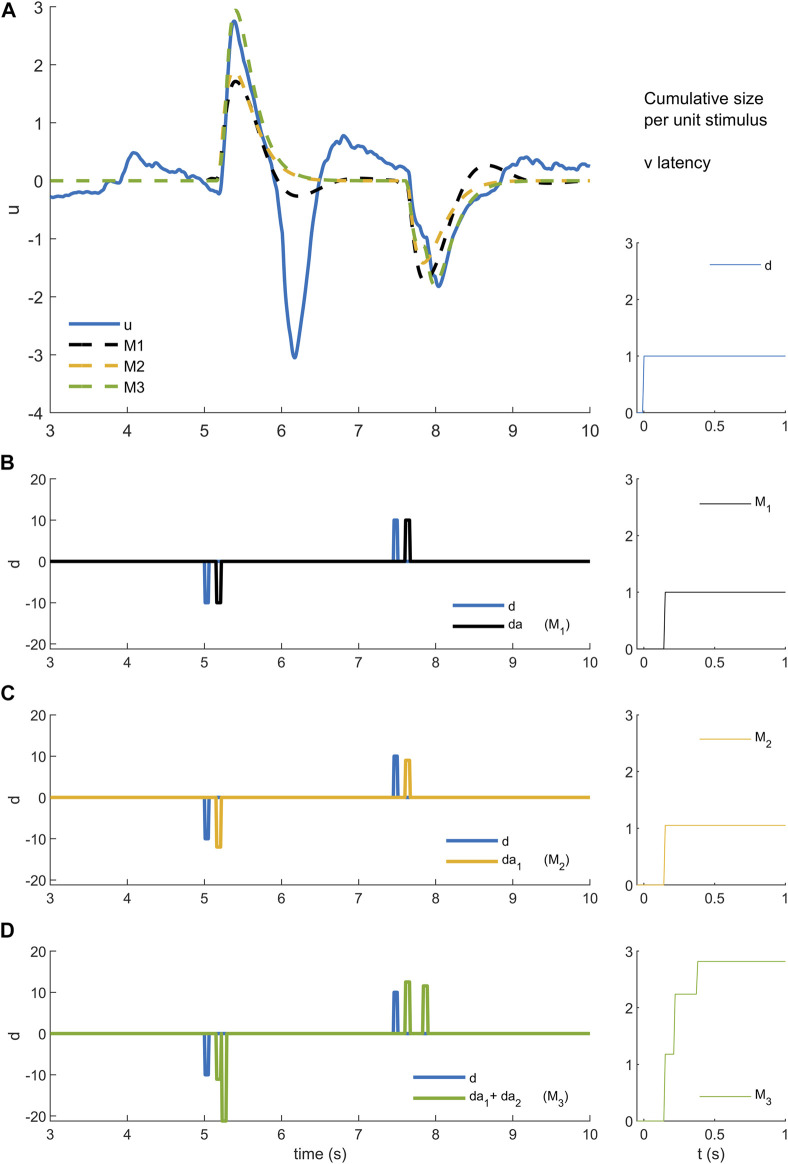
Models M1-3 **(A)**. Experimental control signal u and model (M1-3) simulated control signals. **(B)**. Disturbance d (blue) and model disturbance da (black) for model M1. **(C)**. Disturbance d (blue) and model disturbance da = da1 (yellow) for model M2. **(D)**. Disturbance d (blue) and model disturbance da = da1 + da2 (green)for model M3. Right column. Cumulative size of model simuli versus latency. Rows A-B show experimental stimulus (blue), M1 model stimulus (black), M2 model stimulus (yellow) and M3 stimulus (green)). Message: Non-linearity is modelled by reforming and optimizing the disturbance sequence applied to an ARX model structure. Detail: An ARX model is a general representation of a linear system with historical states stimulated by a disturbance and stimulated by random noise. The ARX model can be thought of as system such as a bell that responds in a characteristic manner by ringing and humming in response to impulsive taps and to noise. Model M1 uses the highest possible order ARX model (equal order numerator, denominator polynomials) selected by the AIC criterion to prevent overfitting. The ARX model is coupled to the experimental disturbance (**B** blue) using a constant delay and constant amplitude. Thus, each stimulus is represented by a model stimulus of constant delay and constant size (**B** black). This model M1 is linear and time-invariant. Model M2 reduces the order of the ARX model to a maximum of 4 and changes the coupling with the disturbance. Model M2 replaces each data stimulus (**C** blue) with a model stimulus of variable amplitude (**C** yellow). The size of the yellow impulse in **C** is individual for each stimulus. For M2, non-linearity lies in the concept that the gain of the response (**A** yellow) can vary stimulus by stimulus. Model M3 limits also the order of the ARX model to a maximum of 4 and uses many parameters to model the coupling between disturbance and ARX. Model M3 replaces each data stimulus (**D** blue) with a model doublet (**D** green) representing a fixed delay “reflex” and later variable delay “voluntary” stimulus. The size is optimised for each stimulus of each doublet. The delay is optimised for the second stimulus in each doublet. For this model, the non-linear concept is that each real stimulus evokes two discrete responses from the motor system. This concept is general: it could represent two parallel pathways i.e., a direct pathway with fixed delay and an indirect pathway with variable delay. The concept could also represent a single pathway with serial, event triggered responses following an initial response at fixed delay. From a temporal sequence of stimuli, the cumulative size shows for each time step t the mean size $s_{\left(t\right)}$ of all n stimuli si occurring at latency less than timestep t where $s_{\left(t\right)}=\sum_{i=1}^{n}\frac{s_{i}}{n}$ . Stimulus amplitude si is in units normalised to the size of the experimental stimulus. The cumulative size for experimental stimulus is zero for t < 0 s and unity for t ≥ 0 s (right column). The cumulative size for model M1 is zero below, and unity above, the fixed delay (right column).

**FIGURE 4:**
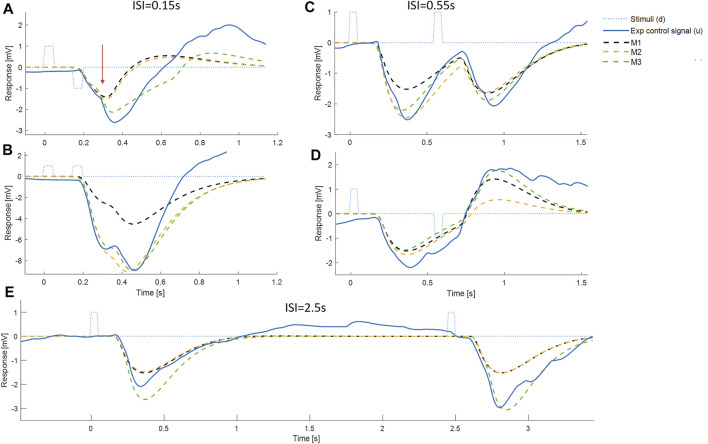
Representative timing and composition of responses to discrete impulses. All panels show: Representative experimental myoelectric control signal (u) (blue solid line), Impulse Stimuli (thin dotted blue lines), Single process, LTI Model M1 (dashed black) Single process model M2 including fixed delay and variable amplitude model (yellow dashed), Two process model M3 allowing a fixed delay response and a variable delay response, each with variable amplitude (green dashed). Note. The measured delay includes a precise “Trigno” delay of 48 ms to the output of EMG. The myoelectric response was produced in the muscle 48 ms earlier than the instant recorded. Message: At short ISI, the experimental control signal shows substantial departure from the LTI (M1) behavior. E.g., panel A, see vertical arrow, the experimental response deviates markedly from LTI in a direction defined by stimulus 1 and not stimulus 2. This experimental non-linearity is captured by the two-process model M3, but not by the single process model M2. At all ISI, the initial onset at ∼0.16s is well represented by the LTI (M1) and single process model (M2). At all ISI, a second onset is visible at ∼0.3 ± 0.1 s.

**FIGURE 5:**
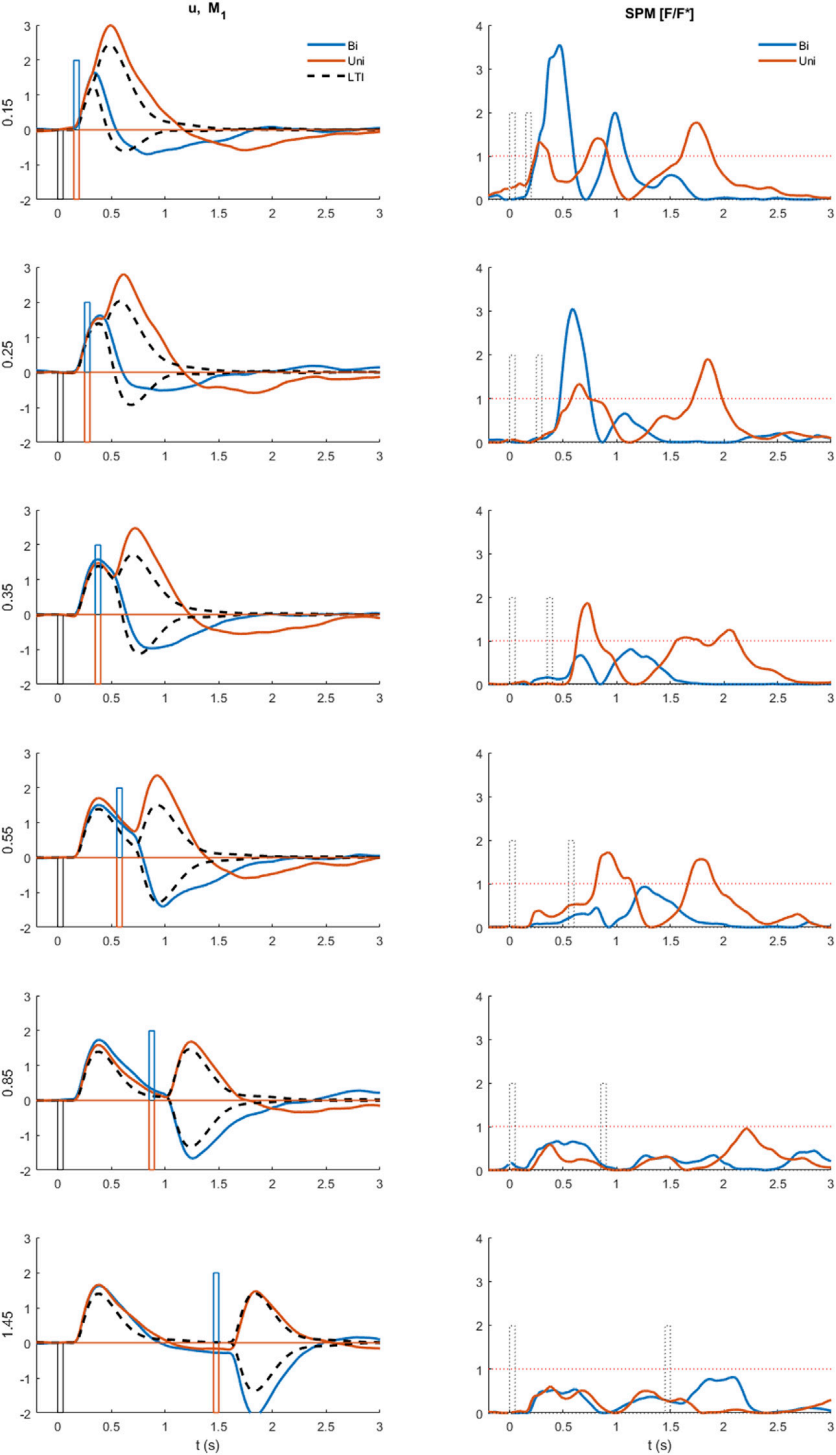
Comparison of experimental control signal u with LTI prediction. Left: Experimental myoelectric control signal (u) for Bi direction (blue) and Uni directional (red) paired stimuli. Single process, LTI Model M1 (dashed black). Impulse Stimuli (rectangular lines). All signals averaged over all cases for each subject, and then averaged over all subjects. N.B. For all positive first stimuli, all signals reversed to align to negative first stimulus and positive first response. Right: Time varying F statistic relative to unity threshold (F/F* at alpha 0.05) for Bidirectional (blue) and Uni directional (red) paired stimuli. (1-d SPM, repeated measures Anova, *n* = 22). Impulse Stimuli (rectangular dotted lines), Message: At short ISI, the experimental control signal shows substantial departure from LTI (M1) behavior.

Initially (Figure 2, 1st question), we test whether the perturbation-response data (d, u) (Figure 3A,B blue lines) are consistent with a linear-time-invariant (LTI) process (Figure 3A black line), or whether at low ISI there are systematic deviations from linearity. We construct a generalized linear-time-invariant (LTI) model to represent the prevailing ideas of balance (van der Kooij and de Vlugt, 2007; Kiemel et al., 2011; van der Kooij and Peterka, 2011; Safavynia and Ting, 2013a; Safavynia and Ting, 2013b; Crevecoeur and Kurtzer, 2018; Kurtzer, 2019). Model M1 (high order ARX) is a single LTI process with fixed delay $\hat{\Delta }$ . We emphasize that ARX, a linear black-box input-output model, is an equivalent model to a linear state-space model (Phan and Lngman, 1996). An ARX model contains historical system states and can be transformed into an equivalent state-space model and vice versa, with no loss of information (Supplementary Figure S1). This generalized model M1 is equivalent in principle to any time delayed, continuous, linear feedback controller including one with or without a state estimator and including one with or without a state predictor (Ljung, 1999; Pintelon and Schoukens, 2001; Gawthrop et al., 2009). Model M1 simulates an experimental control signal from the independent stimulus d. For all ISI’s we compare the experimental control signal u with a model simulated version (Figures 4, 5). To test systematic deviation of the experimental response u from the LTI model M1 simulated response, we use a validated statistical analysis for computing the time varying F statistic (1-d SPM, (Pataky, 2012)) (Figure 5). We test whether the experimental control signal u shows significant, systematic deviation from linearity at low ISI.

Second (Figure 2, Qu’s. 2, 3), we model (non-parametrically) two non-linearities for their potential to represent and explain any systematic deviation from continuous LTI behavior at short ISI.

Model M2, a single non-linear process (fixed delay $\hat{\Delta }$ , time-varying amplitude), implements the physiologically supported idea that gain of the long-latency reflex can vary according to task level goal, intention and whole body state (Pruszynski and Scott, 2012). Model M2 adds to model M1 an individual amplitude parameter for each stimulus. Each stimulus is applied at fixed delay $\hat{\Delta }$ , same as model M1. Optimization of each amplitude gives an estimate of the response amplitude to each stimulus (Figures 3A,C yellow).

Model M3, two non-linear processes (one fixed delay $\hat{\Delta }$ , one variable delay Δ, each of time-varying amplitude), implements the physiologically plausible idea that a second process contributes to the balance response at a variable latency later than the transcortical delay (Marsden et al., 1981; Marsden et al., 1983). Model M3 adds to model M2 an additional stimulus with an individual amplitude parameter and individual delay for each stimulus. Optimization of each delay and amplitude gives an estimate of the response amplitude and delay to each stimulus for the second process (Figures 3A,D green).

**FIGURE 6:**
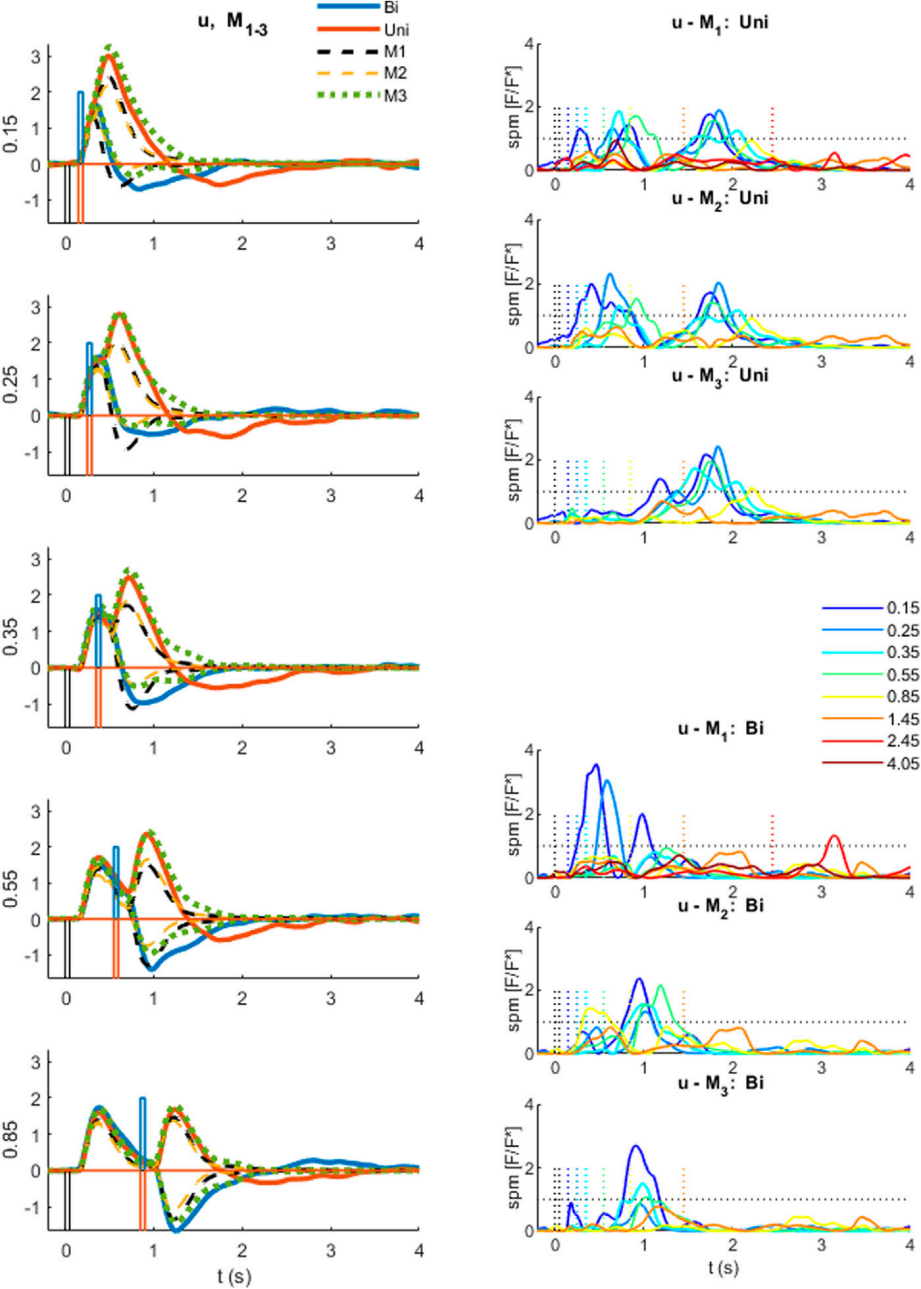
Comparison of experimental control signal u with models M1-3. Left: Experimental myoelectric control signal (u) for Bidirectional (blue) and Uni directional (red) paired stimuli. Single process, LTI Model M1 (black dashed). Single process varying amplitude model M2 (yellow dashed). Two process (fixed and variable delay) model M3 (green dotted). Impulse Stimuli (rectangular lines). All signals averaged over all cases for each subject, and then averaged over all subjects. N.B. For all positive first stimuli, all signals reversed to align to negative first stimulus and positive first response. Right: Time varying F statistic relative to unity threshold (F/F* at alpha 0.05) for all ISI intervals. Uni directional (rows 1–3) and Bidirectional pairs (rows 4–6). (1-d SPM, repeated measures Anova, *n* = 22). Impulse Stimuli (rectangular lines), Message: At short ISI, the two-process model M3 reproduces the experimental control signal u most closely.

Using the same time-varying statistical analysis (1-d SPM) we investigated systematic deviation from the experimental control signal u, at all ISI. The objective of models M1-3 is to capture without systematic error the experimental response for all ISI (Figures 4–6).

**FIGURE 7:**
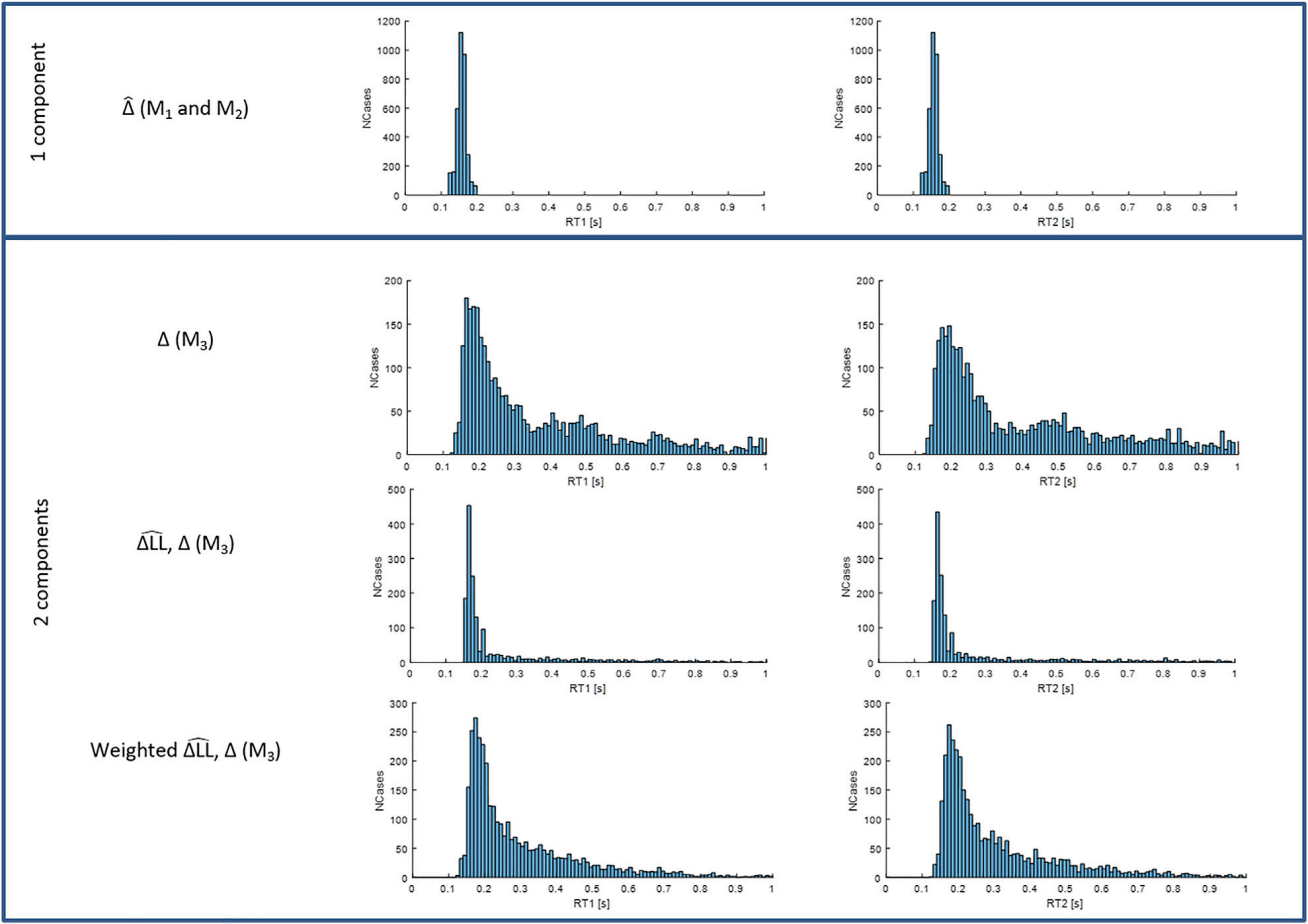
Distributions of RT1 (first column) and RT2 (second column) for models M1-3. Each subplot shows the number of cases (*y*-axis) for each RT value within the physiological range (*x*-axis). Row 1: fixed delay $\hat{\Delta }\;$ for models M1 $\left(\hat{\Delta }\right)$ and M2 $\left(\hat{\Delta }\right)$ . Row 2: variable delay Δ from two process model M3 ( $\hat{\Delta LL}$ , Δ ). Row 3: fixed delay $\hat{\Delta }\;$ and variable delays Δ from model M3 ( $\hat{\Delta LL}$ , Δ ) in the same histogram. Row 4: integrated delay averaging each delay with a weight equal to its size from model M3 ( $\hat{\Delta LL}$ , Δ ). Message: A two process model shows the response time is much longer than revealed by a single process model.

**FIGURE 8:**
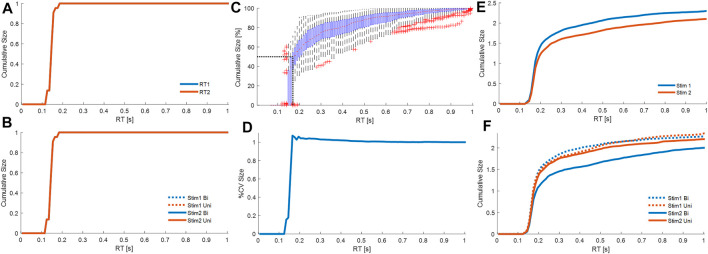
Cumulative contribution at each latency. Cumulative size of the response from all processes M1. Combining all participants, and all trials from each participant, panels show cumulative size of fixed delay responses curves at latency (0–1 s). **(A)**: cumulative absolute size for stim1 (blue) and stim2 (red) relative to the stimulus size. **(B)**: cumulative absolute size for Uni (red) and Bi (blue) directional pairs; stim1 (dotted), and stim2 (solid). Cumulative size of the response from all processes M3. Combining all participants, and all trials from each participant, panels show cumulative size of fixed delay and variable delay. **(C)**: cumulative size (% of trial value at 1s, all cases normalized per trial) vs. latency. Boxplots for each latency show all trials. The red dots are median values between trials. Red crosses are outliers. Horizontal and vertical dotted black lines respectively indicate 50% total cumulative size and the corresponding latency (185 ms). **(D)**: cumulative coefficient of variation (%) vs. latency. **(E)**: cumulative absolute size for stim1 (blue) and stim2 (red) relative to the stimulus size. **(F)**: cumulative absolute size for Uni (red) and Bi (blue) directional pairs; stim1 (dotted), and stim2 (solid). Panels A–B, D-F show average of all participants, where each participant is an average of all their cases. Message: Strength of response and function differ between fixed and variable delay processes.

**FIGURE 9:**
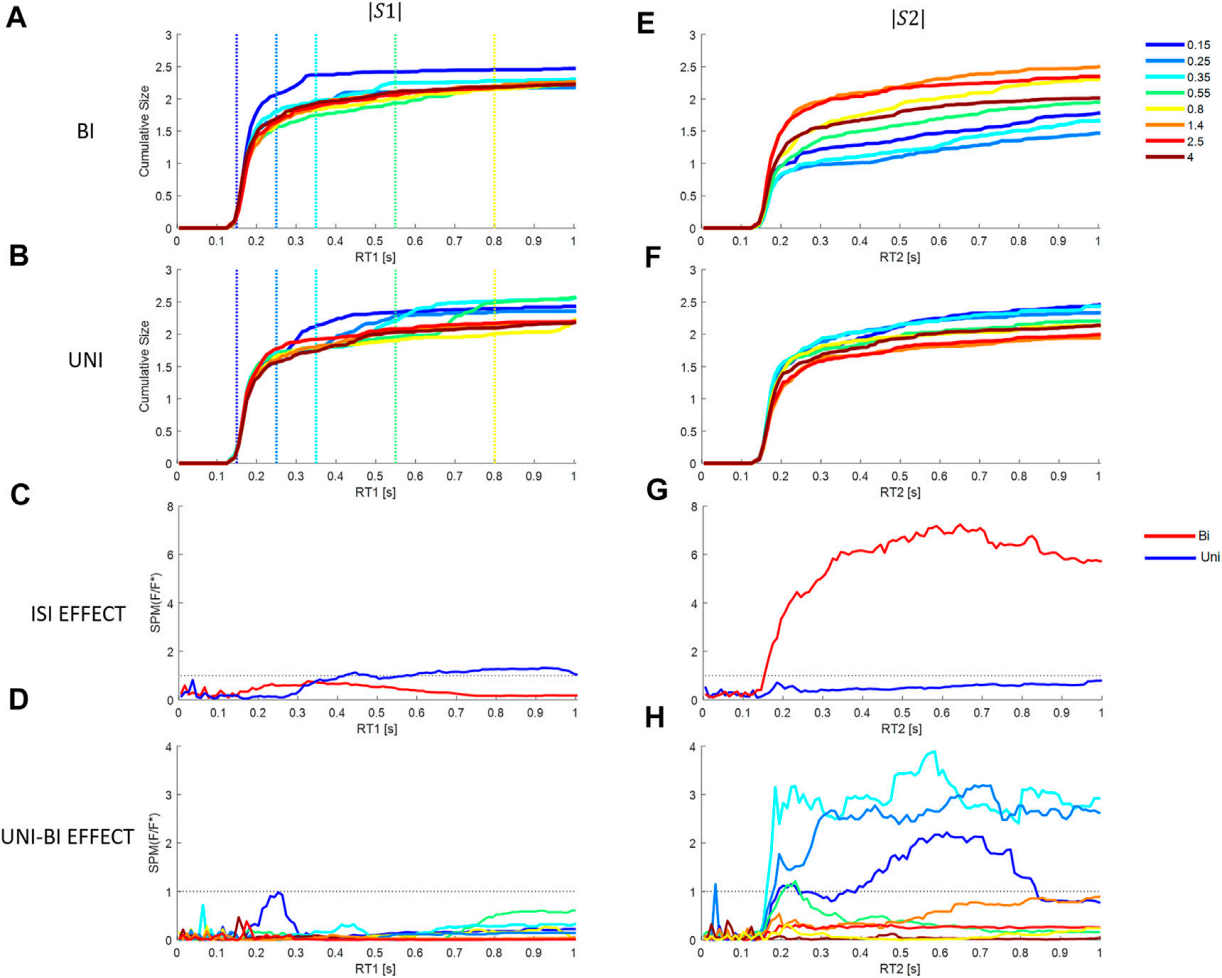
Effect of Inter stimuli interval and direction on cumulative size response from model M3 at each latency. First column (stim1), second column (stim2). Rows 1 and 2: Cumulative response size for each ISI (colors in the bottom right legend) relative to the stimulus size. Row 1 (A, E): Bidirectional pairs. Row 2 (B, F): Uni directional pairs. Row 3 (C, G): SPM Time varying (F/F* at alpha 0.05): Effect of ISIs at each latency. Bi (Red) and Uni (blue). Row 4 (D, H): SPM Time varying (F/F* at alpha 0.05): Effect of paired direction (Bi v Uni). Colors show ISIs. Rows 3–4: Horizontal dotted line shows F/F* = 1 (threshold required for significance difference at alpha = 0.05). Message: Non-linearity: triggered reactions or refractoriness is revealed by significant effect of ISI.

Using model, M3, which captures the response u at all ISI, we investigated the cumulative temporal distribution of discrete balance responses and hence the contribution of fixed delay and variable delay components to the experimental response (Figures 7, 8). Using time varying statistical analysis (1-d SPM) we studied the effect of stimulus order (1 v 2), stimulus direction (Uni v Bidirectional) and ISI (0.15, 0.25, 0.35, 0.55, 0.85, 1.45, 2.45, 4.05s) on the cumulative amplitude v time of discrete responses of both processes from model M3 (Figures 8, 9). These analyses provide new evidence of the strength and function of the slower contributions to the balance response.

## Materials and methods

### Ethical approval

The experiments reported in this study were approved by the Academic Ethics Committee of the Faculty of Science and Engineering, Manchester Metropolitan University (Ethos Ref. 0,567) and conform to the Declaration of Helsinki. Participants gave written, informed consent to the experiment which was performed in the Research Centre for Musculoskeletal Science & Sports Medicine at Manchester Metropolitan University. Participants in videos gave consent for publication.

### Balance task

This apparatus and task have been reported previously in detail and provides balance control data defining a current benchmark for quality (Cherif et al., 2020; Loram et al., 2022). In brief, participants stood with their feet on a stable base and used their own muscles and their own natural senses to maintain balance of their own body while strapped rigidly to a one degree of freedom actuated device, named Whole Body Mover (WBM) (Figure 1, Supplementary Videos S1, S2). This approach allows precise measurement of the disturbance d, the control signal u and system output (position), and also provides a known external system, and a known neuromuscular system converting EMG into force. In natural standing the control signal for a multi-segment system is hard to define, the neuromuscular and mechanical system are also hard to define precisely, and system output (whole body CoM) is difficult to measure precisely. In natural balance, separation of neuromotor from passive contributions to the control signal is imprecise.

The WBM is composed of a vertical board rotating around a joint collinear with the ankles, connected to a direct drive linear actuator at approximately 1 m above the axis of rotation. As published previously (Cherif et al., 2020; Loram et al., 2022), the control signal u applied as net torque to the WBM was generated by a myoelectric interface sampling plantar flexion and dorsi-flexion action of both calf (soleus + gastrocnemius medialis) and tibialis anterior muscles (see Supplementary Appendix A1 for detail). The EMG system (Trigno, Delsys Inc., Boston, United States) imposed a precise digital delay of 48 ms to all EMG signals. The 2nd order dynamics of the WBM were set to ensure the closed loop system replicates the temporal dynamics, sway distribution of natural postural balance (Loram et al., 2022). The WBM becomes the body of the participant to be controlled with their postural leg muscles. The sensory feedback, the motor action the ownership of self-movement and distribution of sway size and speed ensure the task feels natural and very similar to postural balance ((Loram et al., 2022) and Supplementary Video S2).

### Participants and experimental protocol

Twenty-two healthy participants (7 F + 15 M, 35 ± 11 years) took part in the experiment. Participants were prepared for the myoelectric interface. Participants were then given a short familiarization with the task without using perturbations for approximately 5 min which was sufficient to feel comfortable with the task. Then additional force stimuli were provided (Figures 1B,C): participants were told that every now and then, the WBM would gently push them forwards or backwards and were instructed to keep the WBM within apparatus limits (±10°) which exceeded typical unperturbed sway by an order of Magnitude. Each participant performed 5 trials of 250 s duration.

### Experimental design and statistical analysis

Stimulus Design: We chose a discrete stimulus d that would evoke a clear balance response in the myoelectric control signal u (Figure 1C). Each individual stimulus was an impulse of constant force lasting 50 ms evoking a distinct acceleration and following a physiological delay a myoelectric response (Figure 1C). The sequence of impulse stimuli was designed to test variability in delay and size of the response, and the effect of inter-stimulus-interval (ISI). For each trial, 32 pairs of impulses were selected randomly from a set of eight levels of ISI (0.15, 0.25, 0.35, 0.55, 0.8, 1.4, 2.5, 4 s), with all four stimulus direction combinations for each level (forward-forward, forward-backward, backward-forward, backward-backward) (Figure 4). Following each paired stimulus, there was uniform distribution of random recovery periods ranging 4–6 s.

Estimation of stimulus-response parameters: We fit three stimulus-response, time-series models (M1, M2, M3) (Figure 3). Model M1 (ARX) is a single linear-time-invariant (LTI) process with fixed delay. Model M2, a single non-linear process (fixed delay, time-varying amplitude), adds an optimized response amplitude to each stimulus. Model M3, two non-linear processes (one fixed delay, one variable delay, each of time-varying amplitude), add a second process of optimized delay and optimized response amplitude to each stimulus.

Figure 2 shows the sequential testing of Questions 1-4 to test our hypothesis (H) that the fixed delay pathway provides the minor contribution to balance.

Testing for significant differences between time varying signals at each time-step involves multiple tests. Using an uncorrected ‘F’ statistic at each timestep assumes independence between time steps and incurs the risk of declaring significant differences by chance. Applying a Bonferroni correction for multiple tests assumes no independence between timesteps and risks obscuring differences which are significant. 1-d SPM, which has been validated and applied currently by ∼300 papers (Pataky, 2012), uses random field theory to compute the degrees of freedom in the timeseries and thus to normalize appropriately the time varying ‘F’ statistic.

Qu. 1 Does the experimental control signal u deviate significantly from a LTI process (M1) at short ISI? (Figures 4, 5). We used one dimensional statistical parametric mapping (1-d SPM) (Pataky, 2012) with repeated measures anova to test the time varying within participant effect of model (M1 simulation v experimental) on the ISI windowed control signal u (Figure 5).

Qu. 2. Does time varying amplitude (M2) or a second variable delay, variable amplitude process (M3) account for non-linear behavior of the control signal u at short ISI? We used 1-d SPM (Pataky, 2012) with repeated measures anova to test the time varying within participant effect of model (M2, M3 simulation v experimental) on the ISI windowed control signal u (Figure 6).

Qu. 3 How is response size and function distributed between fixed and variable delay processes?

Using model M3, we assessed the relative magnitude of fixed delay and variable delay processes to the observed response. Model M1 has 62 responses to stimuli of fixed size and fixed delay. Model M3 optimizes the size of 64 fixed delay and 64 variable delay responses for each trial. Size is reported in units relative to the size of stimulus. For each subject (*N* = 22), for each ISI (8 levels), for each directional pairing (Bi or Uni), and for each stimulus (1 or 2), all responses (fixed delay, variable delay) are collated. For each category of participant, ISI, directional pairing, and stimulus number, at each possible latency t in steps of 0.01s up to 1 s, we sum the size of all responses with latency between 0 s and t and divide by the number of stimuli (Figure 8). This process generates 704 (22 × 8 × 2 x 2) empirical curves of cumulative size v latency (Figure 8). For visualization (e.g., Figure 8), we show the relevant mean cumulative size curve. For statistical analysis we used one dimensional statistical parametric mapping (Pataky, 2012) with repeated measures anova to test the variation with latency of the within participant effect of stim (1 v 2) on cumulative size and then to test the within participant effect of direction (bi v uni) within each stimulus category (1 or 2) (Figure 8).

**FIGURE 10:**
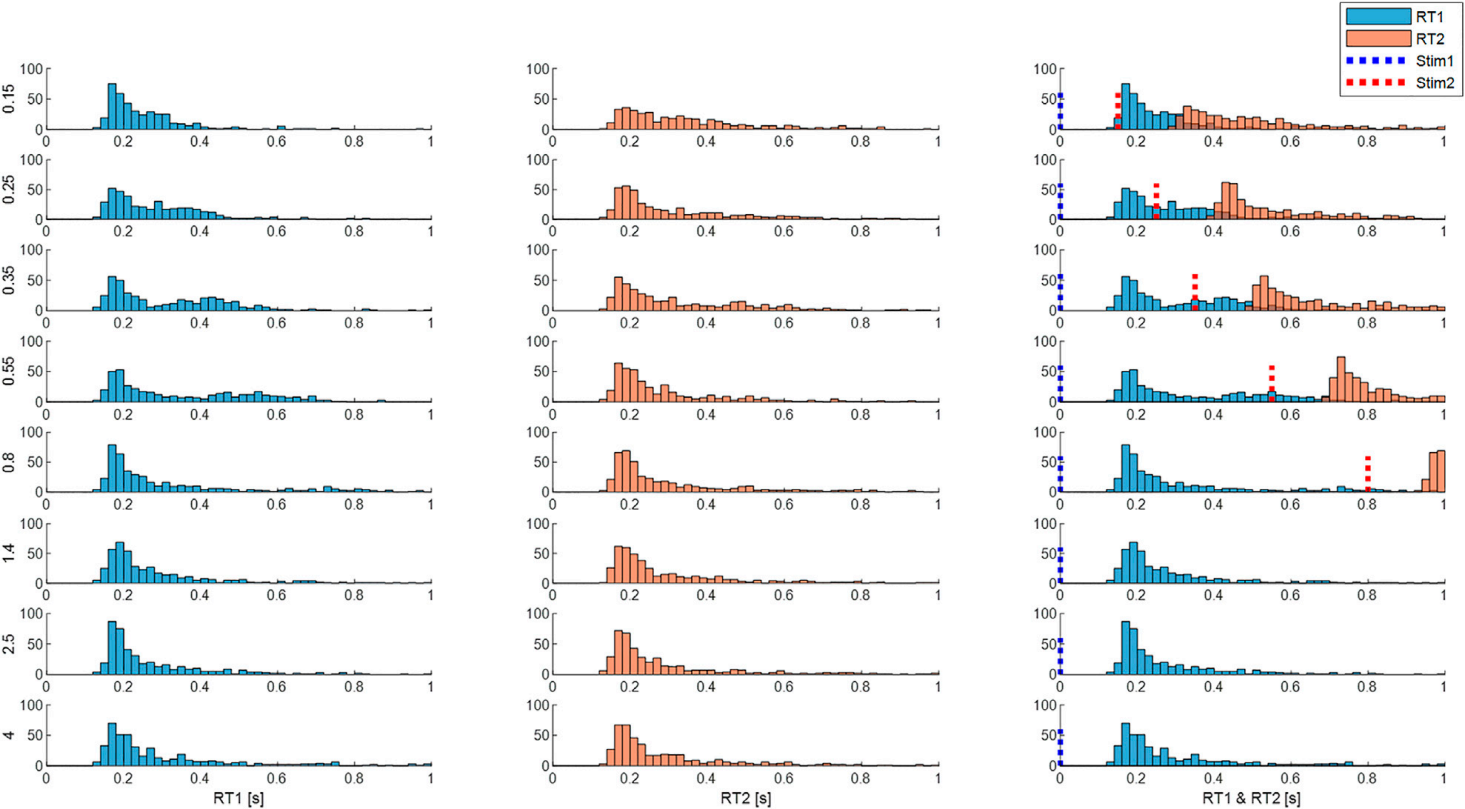
Response times are affected by inter-stimulus interval (ISI). Panels show distribution of size integrated delays from M3 (both processes). Rows: ISI 0.15—4 s. Column 1 (RT1). Column 2 (RT2). Column 3 (RT1 and RT2 related to stimulus 1 onsets). Blue dotted line represents the onset of the first stimuli and the red line represents the onset of the second stimuli. Each subplot shows the number of cases (*y*-axis) for each latency (*x*-axis). Each row shows distribution for each ISI (vertical left label 0.15–4 s). Message: RT1 and RT2 distributions are similar at large ISI. At short ISI, RT2 distribution is longer and flatter.

**FIGURE 11:**
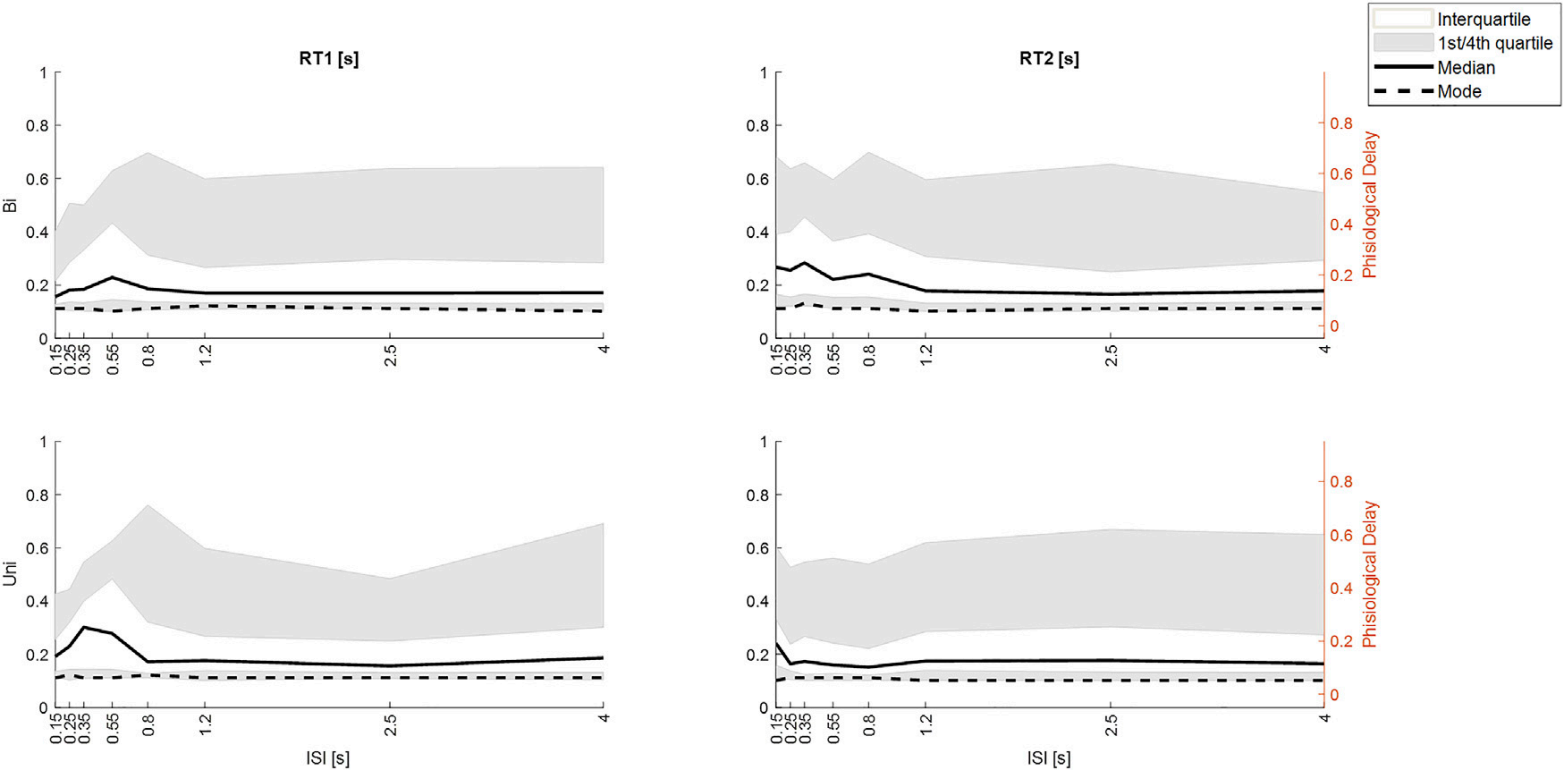
Effect of ISI on response latency differs by stimulus direction history (Uni v Bi). Distribution of integrated response times from M3. Row 1. Bi-directional pairs. Row 2. Unidirectional pairs. Column 1: RT1. Column 2 RT2. All panels: size integrated response time (*y*-axis) v ISI (*x*-axis) the ISIs. Percentiles: white area (interquartile, grey area (1st and 4th quartiles), Dashed line (mode), solid line (median). Given the ∼50 ms delay within the Trigno-EMG system, we show observed delay left *y*-axis and physiological latency of muscle activation (right *y*-axis). Message: Large ISI, RT1 and RT2 are similar for Uni and Bidirectional pairs. At small ISI: RT2 latency increases for ≥50th percentile for Bidirectional pairs at ISI<1.25 s but only for ISI 0.15 for Uni directional pairs.

Qu. 4 Is the cumulative response independent of ISI? To investigate the presence of time variant processes and refractoriness, we test the effect of inter-stimulus-interval (ISI) on curves of cumulative size v latency (Figure 9) and on response delay (Figures 10, 11). We used one dimensional statistical parametric mapping (Pataky, 2012) with repeated measures anova to test evolution with latency of the within participant statistical effect of ISI on cumulative size (Figure 9), and then the within participant effect of direction (Bi v Uni) at each ISI. We report the distribution of response delays to stimulus 1 and 2 (RT1, RT2). Model M3 estimates 64 single identical value delays and 64 unconstrained response delays for each trial. For each stimulus we computed a single integrated delay averaging the delay of each response with a weight equal to its fractional size. A linear mixed effects model with fixed factor ISI (8 levels), random factor ISI, and intercept each grouped by Subject was used to test the fixed effect of ISI on integrated response delay from model M3. We used the Satterthwaite approximation to compute degrees of freedom for the F statistic. A linear mixed effects model with fixed factor Model (3 levels), random factor Model and intercept each group by Subject was used to test the effect of Model on variance accounted for by the model. Variance accounted for is 100 x (1-normalised root mean square remnant).

### Details of time series analysis

Model M1: linear time invariant (LTI) model of the closed-loop balance system (Figures 3A,B).

For each trial, the independent disturbance d and myoelectric closed-loop control signal u (Figure 1B) was used to estimate a time-series model (ARX with minimal forward one step prediction error, timestep 10 ms, autoregressive in myoelectric control signal (u) with exogenous input disturbance d including a dead-time). M1 uses an ARX structure (A, B polynomials of equal order q with no delays) $A_{\left(q\right)}u\left(t\right)=B_{\left(q\right)}d_{a}\left(t\right)+e\left(t\right)$ to model evolution through time t of the experimental control signal u subject to an analytic model disturbance input da and random noise of unit variance e. Physiological delay $\hat{\Delta }$ is represented in the model disturbance input da constructed by summation of Nstim stimuli presented at times tistim where $d_{a}\left(t\right)=\;\sum_{i=1}^{Nstim}\delta \left(t-t_{stim}^{i}-\;\hat{\Delta }\right)*R\left(t\right)\;$ where $\hat{\Delta }$ is a single fixed delay for the trial, δ is the Dirac delta function, R is the rectangular function of unit height and duration 50 ms and ∗ represents convolution (Figure 3).

Using Akaike’s Information Criterion (AIC), the dead-time $\hat{\Delta }$ was selected from range 0.09–0.4s using an 8th order model. Next, using AIC, the model order (q) was selected limited to a maximum of 40 to enable a high order LTI model. Next the model disturbance input da was created by shifting the time of each sample of disturbance d back by the dead-time $\hat{\Delta }$ and the dead-time of the ARX model was set to zero (Figure 3B). This data (da, u) and the zero delay ARX structure was used to estimate model M1.

This model M1 provides a high order linear-time-invariant (LTI) reference for the following models M2 and M3 used to capture non-linear aspects the response (Figures 3A,B).

### Non-linear models

The Estimation of models M2 and M3 (below) uses a variation of the method published previously (Loram et al., 2012).

Model M2: single process, time varying amplitude (Figures 3A,C).

M2 uses the same ARX structure as above $A_{\left(q\right)}u\left(t\right)=B_{\left(q\right)}d_{a}\left(t\right)+e\left(t\right)$ but non-linearity is represented in optimization of the stimulus sequence da where $d_{a}=\;d_{a_{1}}\left(t\right)=\;\overset{Nstim}{\underset{i=1}{\sum }}s_{i}\delta \left(t-t_{stim}^{i}-\;\hat{\Delta }\right)*R\left(t\right)\;$ where $\hat{\Delta }$ is the same single fixed delay as model M1 and $s_{i}$ is an adjustable size for each stimulus (Figure 3C).

Using AIC, a lower order (q) LTI model was selected, limited to a maximum order of 4 to ensure tractable computation time for the following procedures and initial coefficients for ARX model M2 were estimated. Then the size $s_{i}$ of each impulse in the model disturbance da was optimized iteratively to minimize the forward prediction error of the ARX model M2.

ARX coefficients for the whole trial were estimated afresh at each evaluation of the forward prediction error using size $s_{i}$ . The impulse sizes were optimized sequentially from last stimulus to first stimulus (each with scale factor 0–100) until there was no further improvement. The sequential optimization of 64 sizes was iterated 5 times. For each trial, this analysis yielded 64 size parameters representing the size of response to each stimulus, one delay and one set of 4th order ARX coefficients. Model M2 is intended to represent function (fixed delay, adjustable amplitude) of the reflex pathway.

Model M3: two process: fixed delay and variable delay, each of time varying amplitude (Figures 3A,D).

M3 uses equation $A_{\left(q\right)}u\left(t\right)=B_{\left(q\right)}d_{a}\left(t\right)+e\left(t\right)$ where $d_{a}=\;d_{a_{1}}+d_{a_{2}}$ such that the fixed delay reflex component is $d_{a_{1}}\left(t\right)=\;\sum_{i=1}^{Nstim}s_{LL}^{i}\delta \left(t-t_{stim}^{i}-\;\hat{\Delta_{LL}}\right)*R\left(t\right)\;$ and the variable delay component is $d_{a_{2}}\left(t\right)=\;\sum_{i=1}^{Nstim}s_{VD}^{i}\delta \left(t-t_{stim}^{i}-\;\Delta^{i}\right)*R\left(t\right)\;$ .

Fitted parameters include $s_{LL}^{i}$ and $s_{VD}^{i}$ the adjustable sizes of the long-latency reflex and variable delay response to each stimulus and include $\Delta^{i}$ the adjustable delay for the variable delay response to each stimulus.

As represented in the equation, each impulse in model disturbance da was replaced with two impulses each with an adjustable size (Figure 3D). The time of the first impulse was fixed at the dead-time of the initial model M1 intended to represent the reflex deadtimes. The time of the second impulse was adjustable in the range model M1 deadtime plus one timestep up to 1 s relative to real stimulus. As before, using AIC, a lower order (q) LTI model was selected, limited to a maximum order of 4 to ensure tractable computation time for the following procedures and initial coefficients for ARX model M3 were estimated. The size of each impulse and the time of each variable delay impulse in the model disturbance was optimized individually to minimize the forward prediction error of the model M3. As above, working from last to first stimulus, each stimulus response (two processes) was optimized sequentially until there was no further improvement limited to 5 iterations of the complete trial. This analysis yielded 64 sizes at reflex latency, and 64 sizes and 64 delays at variable latency. Model M3 is intended to represent combined function of both reflex (transcortical) and slower variable delay (voluntary) components of the response to each stimulus.

## Results

This study investigates the closed-loop response of the balance control signal u to independent, discrete, impulsive stimuli d (Figure 1). The impulsive force stimuli d produced experimental accelerations substantially larger and more sudden than those present during background sway (Figure 1C). These stimuli d evoked clear, discrete responses in the control signal u (“Exp response”, Figure 1C). Following the order of questions shown in Figure 2, using three models (M1, M2, M3) we report results in Figures 3–11.

### Non-parametric model statistics

Models M1 to M3 used increasing numbers of parameters to incorporate more of the experimental control signal u within the simulated response to the independent disturbance d and reduce the remnant (Table 1). Validity is determined by absence of systematic error between model simulation and experimental signal at all inter-stimulus-intervals (ISI) (Figures 3, 4). Between models M1 to M3 there was significant increase in variance accounted for (Variance %Fit) (F (2, 37.5) = 27.2, *p* = 0.00000005) with a significant post hoc difference M3—M1 (*p* < 0.0001, using Bonferroni correction) and no significant difference M2—M1 (Table 1). The one step ahead prediction error fit, which includes simulation and remnant within the prediction was high for all models with no difference between models M1-3 (Table 1).

**TABLE 1:** Model statistics.

Model	Description	Variance %fit	Pe %fit	N Params
M1	Linear High-Order ARX	0.133 ± 0.1	99.1 ± 0.2	32.6 ± 20
M2	Non-linear Reflex	0.150 ± 0.09	99.1 ± 0.2	71.6 ± 0.5
M3	Non-linear Reflex + Voluntary	0.215 ± 0.1	99.1 ± 0.2	200 ± 0.5

For models M1-3, columns show mean ± S.D. for percentage of the variance accounted for by the model simulated response, one step ahead prediction error and total number of model parameters.

Methodological note regarding experimental delays: Latencies of the human experimental response u (Figure 1C) are calculated and reported relative to the onset of the virtual force stimulus d. The experimental delay includes a precise “Trigno” delay of 48 ms to the output of EMG signals arising from the EMG system (Trigno, Delsys Inc., Boston, United States). This 48 ms delay conveniently equals the physiological delay (49.7 ± 7 ms) between onset of EMG and onset of force during voluntary (as opposed to electrically stimulated) contraction (Begovic et al., 2014). Thus, the physiological latency of muscle activation, i.e., the latency at which the myographic response was produced is approximately “50 ms”, earlier than the instant recorded and reported. However, the observed latency of the experimental control signal u is correct physiologically for the onset of force generation.

Representative time series (Figure 4) show that responses to stimuli have a clear first onset followed by discrete secondary onsets. There is a consistent, distinct first onset to changes in the myoelectric control signal u at ∼160 ms (Figure 4, all panels, solid blue line). Closer inspection of all panels shows a secondary onset in response to the first stimulus at 250–400 ms (c.f. inflexion in solid blue line) giving a second response component of similar or larger size than the initial component. In panel E (long ISI) a second component is ambiguous for the first stimulus and clearer for the second stimulus and in the correct direction for both stimuli. In panel A (short ISI) the second component (onset indicated by vertical arrow) is large and crucially in the correct direction for the first stimulus and incorrect direction for the second stimulus. In panel D (medium ISI), the second component is large and in the correct direction for the first stimulus. In panel B (short ISI), the second component is present and in the correct direction for the first and second stimuli.

Qu 1. Does the experimental control signal u deviate significantly from a LTI process (M1) at short ISI?

The LTI model M1 expresses the currently accepted closed-loop control model of balance represented by continuous, linear feedback. The LTI model M1 predicts responses at short ISI (Figures 4A–D) which are a linear superposition of two independent closed-loop responses such as those observed at long ISI (Figure 4E). At short ISI, the LTI response to a second same direction stimulus adds to the first response (Figure 4B). At short ISI, the LTI response to a second bi-directional stimulus is reversed and subtracts from the response to stimulus 1 resulting in a small combined LTI response (Figure 4A). However, for Figure 4A, the representative experimental response to stimulus 2 adds to rather than subtracts from stimulus 1. The direction of the response at 0.3 s, is determined by the direction of stimulus 1, even though stimulus 2 occurred at 0.15 s. This representative example at short ISI illustrates refractoriness, i.e., insensitivity to current stimuli, (specifically the direction but not the occurrence of stimulus 2). For these examples (Figure 4), the two-process model M3, comprising a reflex and a variable delay component, captures the initial onset well and the whole response better than both single process models M1 and M2.

Departure from LTI behavior (model M1) at short ISI was shown systematically by all participants (Figure 5). At long ISI (≥0.85 s, Figure 5, rows 5–6), the experimental control signal, is not significantly different from the LTI response (Figure 5 right shows time varying F-statistic). At short ISI (≤0.55 s, rows 1–4), following a second same direction stimulus (Uni), the experimental response (red) is significantly larger than the LTI superposition of independent responses (M1). Non-linear interference at short ISI (Uni), indicated by the rise in F-statistic (Figure 5 right column), occurs one reaction time (∼0.16 s) after the second stimulus and shows an exaggerated response in the direction defined by stimulus 1. At short ISI (≤0.25 s, rows 1–2), following a second opposite direction stimulus (Bi), the experimental control signal (blue) shows a reduced negation of the response to stimulus 1 compared with the LTI prediction (M1). This non-linear difference from LTI is significant (F-statistic) from 0.1 to 0.2 s after the second stimulus. Following the initial reduced negation of response to stimulus 1, the experimental control signal u, remains larger in magnitude in the direction required by stimulus 2, than is predicted by the LTI model M1 for up to 1s after stimulus 2.

Qu. 2.Does time varying amplitude (M2) or a second variable delay, variable amplitude process (M3) account for non-linear behavior of the control signal u at short ISI?

Model M2 represents ability of the central nervous system to vary the gain of reflexes. Model M2 allows discrete temporal variation of response gain for each stimulus. Model M3 represents addition of a voluntary response to the reflex response. Model M3 adds a second process to model M2 allowing discrete temporal variation of response gain and delay beyond long latency for each stimulus.

At long ISI (≥0.85 s), all models M1-3 represent the experimental control signal u without significant difference (Figure 6): this result is shown by the signals (Figure 6, left row 5) and by the time varying F-statistic (Figure 6, right all rows) for ISI (≥0.85 s).

At short ISI (≤0.55 s, Figure 6 left rows 1–4), following a second same direction stimulus (Uni), the time varying amplitude model (M2) is similar to the LTI model (Figure 6, left) and remains significantly different from experiment (u—M2) (Figure 6, right row 2). However, the two-process model M3 replicates the experimental response without significant difference up to 1s post stim 2, excepting only ISI 0.15 s above 0.85 s post stim 2 (Figure 6, right row 3).

At short ISI (≤0.55 s, Figure 6 left rows 1–4), following a second opposite direction stimulus (Bi), the time varying amplitude model (M2) eliminates the significant difference (u—M2) up to 0.5 s post stim 2 (Figure 6, left) but retains some significant difference (u—M2) after 0.5 s (Figure 6, right row 5). The two-process model M3 replicates the experimental response most closely of all models M1-3, with no significant difference (u—M3) up to 0.6 s for ISI ≤ 0.35 s, and no significant difference (u—M3) up to 1 s post stim 2 for ISI 0.25 s (Figure 6, right row 6). Reduced but significant difference (u—M3) remains at 0.6–1 s (ISI 0.15 s), 0.6–0.7 s (ISI 0.35 s) and 0.35–0.75 s (ISI 0.55 s) post stim 2, with the experimental control signal u larger than M3 in the direction required by stimulus 2.

### The distribution of fixed and variable delays

Figure 7 shows that when the timing and size of all components are included (last row), response delays show a wide temporal distribution up to 600 ms and beyond and with a main peak at 170–180 ms. The LTI model M1 and single process variable amplitude model M2, (Figure 7, 1st row), which both allow a single delay $\hat{\Delta }\;$ for each 250s trial, shows a narrow distribution of delays peaking at 150–160 ms. From the two-process model M3, the variable delay process Δ shows a wide distribution up to 1,000 ms with a main peak at 170–180 ms, a second smaller peak at 450 ms, and a third small peak at ∼700 ms (Figure 7, 2nd row): the fixed delay process not shown is the same as models M1-2. Reporting both processes, the two-process model M3 shows a main peak at 180 ms, and an extended small tail up to 1,000 ms (Figure 7, 3rd row). When both processes of model M3 are weighted according to their size and integrated into a single size-weighted integrated delay, the temporal distribution peaks at 170–180 ms and shows a long tail up to 1,000 ms (Figure 7, 4th row). Statistical analysis (linear mixed effect) shows the mean delay differs between models. Marginal mean delays for RT1 are 152 and 280 ms respectively for M1 or 2 and M3 (size integrated delay) (F1, 22.1 = 165, *p* = 1 × 10–11). For RT2 the corresponding values are 152 and 285 ms respectively (F1, 21.5 = 269, *p* = 1 × 10–13). In summary, the single process fixed delay models M1,2 are not able to reproduce the size integrated distribution of responses revealed by the two-process model M3 (Figure 7).

Qu. 3. How is response size and function distributed between fixed and variable delay processes?

For a linear-time-invariant (LTI) process, discrete, impulsive stimuli each initiate a temporal response pattern of constant size irrespective of the current state or history of the system. In the analysis that follows, we analyze the response of the balance system by the distribution through time of the size of inputs given to the LTI process.

For model M1, representing balance as continuous state feedback with fixed function i.e., a LTI process, impulsive stimuli of constant size equal to the actual experimental stimulus are delivered at a fixed delay $\hat{\Delta }$ with the distribution over trials shown in Figure 7, row 1. Accumulation through time of these constant size inputs at fixed delay $\hat{\Delta }$ , averaged across all trials and all subjects gives the cumulative size of response shown in Figure 8A. Figure 8B shows this cumulative distribution separately for Uni directional pairs and for Bidirectional pairs and for each stimulus (RT1 and RT2). As expected, each distribution overlaps completely and statistical analysis (1-d SPM) confirms no statistical difference between stimulus order (RT1 v RT2), or direction of second stimulus (Uni v Bi). For stimuli of fixed delay, accumulating the response adds no value to the analysis. However, for responses occurring at variable delay, accumulating the response allows statistical analysis at each delay of all preceding contributions. For model M3, two process, one of variable size at fixed delay $\hat{\Delta }$ , and one of variable size at variable delay, both provide input to a LTI process which is constant for the trial. The temporal distribution of these responses is shown in Figure 7 row 3. Accumulation through time of these variable size inputs at fixed and variable delay, gives the cumulative size distribution (Figure 8C), and when averaged across all cases per subject and then all subjects gives the cumulative size response shown in Figures 8E,F. From Figure 6, Model M3 represents the balance response most accurately including systematic non-linearity not included in Model M1. The cumulative size response of M3 (Figure 7) shows the complete balance response differs from the linear model (Figures 8A,B) and develops substantially at timescales beyond the fixed (reflex) delay.

Model M3 with two processes, fixed delay (reflex) and variable delay (voluntary), enables us to investigate the magnitude and function of each process.

Using both processes from M3, the main strength of the response arises from the variable delay rather than the fixed delay processes (Figure 8C). Averaging all participants and accumulating the size of all fixed and variable delay responses to both stimuli, the cumulative response size (percentage of value at 1s) increases from zero at 125 ms, rises rapidly until 150 ms, reaches 50% at 183 ms, and plateaus above 0.6s (Figure 8C). The fixed delay process at reflex latency (150–160 ms) lies below 183 ms and thus provide a minority contribution to the overall response.

The variable delay process is associated with a slight reduction in variability (Figure 8D). Variation in the response shown by cumulative coefficient of variation (CoV) is highest at 165 ms (∼1.07) and decreases beyond 165 ms to ∼1.002 at 0.7 s (Figure 8B). SPM analysis shows no significant effect on cumulative CoV of stim (1 v 2), or of direction (Bi v Uni) within each stim (1 or 2) (SPM{F}<F*α = 0.05 where F*α = 0.05 is the critical value of F at *α* = 0.05).

Fixed delay processes are insensitive to whether a stimulus is first or second, whereas variable delay processes respond differently to first and second stimuli. For stimulus1 and stimulus2, each cumulative size distribution overlaps up to 157 ms (SPM{F}<F*α = 0.05) and differs above 157 ms (SPM{F}>F*α = 0.05) (Figure 8E). Beyond 157 ms, compared with stimulus1, responses to stimulus2 take longer to accumulate to the same size (Figure 8E).

Are fixed delay processes or variable delay processes sensitive to the direction of stimulus 2 relative to stimulus 1? The first stimulus within stimuli pairs is randomly positive or negative. For bidirectional pairs, the second impulsive stimulus negates mechanically the impulsive input provided by the first stimulus. For unidirectional pairs, the second impulsive stimulus adds mechanically to the impulsive input provided by the first stimulus.

Refractoriness. Response to stimulus 1: The fixed and variable delay response to stimulus 1 is not affected the arrival of a second stimulus. Following stimulus1, the second stimulus arrives at various inter-stimulus intervals during the response to stimulus1. The cumulative size distribution for stimulus 1 shows no difference between uni and bidirectional pairs (Figure 8F). For all latencies up to 1s, the uni and bidirectional cumulative size curves overlap and SPM analysis confirms no significant effect of direction (Bi v Uni) (SPM{F}<F*α = 0.05).

Response to stimulus 2: The fixed delay process is not sensitive to the direction of stimulus 2 relative to stimulus 1, whereas the variable delay response to stimulus 2 varies depending upon the direction of the preceding stimulus 1. For responses to stimulus2, the first and second stimulus arrive before onset of the response to stimulus2. Inspection of Figure 8F shows the cumulative size response to stimulus2 is the same for Bi and Uni directional pairs below 174 ms. SPM analysis confirms significant difference (Bi—Uni) only above 174 ms (SPM{F}>F*α = 0.05).

These results differentiate variable delay from fixed delay process. The variable delay process contributes most of the strength and is sensitive to more preceding information than the fixed delay process.

Qu. 4 Is the cumulative size response independent of ISI?

Inter-stimulus-interval (ISI) has no influence on a LTI process such as model M1. SPM statistical analysis confirms no effect of ISI, or direction (Uni v Bi) at any ISI, on the cumulative size response of M1. These curves are not shown since they are identical to Figures 8A,B for each ISI and each directional pair (Uni, Bi). Below, we report the cumulative size response of model M3 which captures the systematic non-linear behavior without significant error up to 0.6 s (Figure 6).

We used paired stimuli with eight inter-stimulus-intervals (ISI), ranging 0.15–4 s, to test for non-linear processes such as refractoriness and triggered reactions.

Refractoriness features: For responses to stim1, refractoriness is the continuation of a response defined by stimulus1 without modification by the arrival of stimulus2. Modification of the response to sim1 following stim 2 becomes possible after a latency given by the ISI plus some physiological delay (∼0.15 s) and would be evidenced by an effect of ISI, and more specifically by an effect of direction (Uni v Bi) at each ISI. Triggered reactions: By triggered reactions we mean exciting by the arrival of stim2, an additional pre-prepared response to sim1 in the correct direction for stimulus1, irrespective of the direction of stimulus 2. Triggered reactions are refractory to the direction of stim2 but not the occurrence of stimulus2.

For responses to stim2, refractoriness, i.e., lack of responsiveness, is a non-linear suppression or increased delay of responses to stim2. Refractoriness occurs at short ISIs when processing of a current stimulus requires completion of a serial process initiated by responding to a previous stimulus. For stim2, refractoriness is indicated by a significant effect of ISI. We report results separately for unidirectional and bidirectional pairs of stimuli.

Response to Stimulus 1: For responses to stimulus1, arrival of a second stimulus causes no significant effect of direction (Bi—Uni) at any ISI (Figure 9A v Figures 9B,D) (SPM{F}<F*α = 0.05). The response to stimulus 1 is insensitive to the direction of the second stimulus after the second stimulus arrives. For bidirectional stimulus pairs, SPM statistical analysis confirms no effect of ISI at any latency (Figure 9C) (SPM{F}<F*α = 0.05).

For unidirectional pairs, SPM analysis confirms a significant effect of ISI beyond 0.417 s (Figure 9C) (SPM{F}>F*α = 0.05). This effect of ISI on response to stimulus 1 in unidirectional pairs confirms departure from LTI behavior. The non-linear effect of the arrival of stimulus 2 is to increase the size of response in the direction of stimulus 1 (and stimulus 2) (Figure 9B).

Response to Stimulus 2: Statistical analysis of bidirectional pairs confirms a highly significant effect of ISIs beyond 153 ms peaking at 0.65 s for response to stimulus2 (SPM{F}>F*α = 0.05) (Figures 9E,G). The non-linear effect of ISI (at ISI <0.8 s), is to reduce the cumulative response to stimulus 2 at latencies beyond 153 ms. For unidirectional pairs, there is no effect of ISI on response to stimulus2 (Figure 9G) (SPM{F}<F*α = 0.05), though the tendency is to increased response at short ISI (Figure 9F).

For all ISI ≤0.35 s, the effect of direction (Uni v Bi) is significant at latency 162–180 ms onwards and for ISI 0.55 s, the effect of direction is significant at 210–250 ms (SPM{F}>F*α = 0.05, Figure 9D. The non-linear suppression of response to stimulus 2 in relation to direction relative to stimulus 1 (Bi—Uni, Figure 9E v Figure 9F) occurs at short ISI only (≤0.55 s): this non-linear suppression is evident at variable delay beyond 160 ms and the effect increases to a maximum at ∼0.6 s (Figure 9H). For ISI beyond 0.55 s there is no significant effect of direction (Bi-Uni, Figure 9H).

Summary answer to question 4: there is evidence of non-linearity related to ISI and of refractoriness. Refractoriness is revealed by insensitivity of ongoing responses to stimulus 1 to the direction of stimulus 2. Response to stimulus 1 is refractory to the direction but not the occurrence of the second stimulus (Figures 4A, 9B,D). Refractoriness is revealed by the non-linear suppression of response to stimulus 2 at short ISI ≤0.55 s and when stimulus 2 is in the opposite direction to stimulus 1 (Figures 9E,G). A linear-time-invariant response to stimulus 2 would overlap at all ISI and for all directions (Uni v Bi).

### The temporal distribution of responses to independent and interfering stimuli

Using size-weighted integrated delay to summarize the central latency of the response to stimulus from both processes of model M3, Figure 10 combines Uni and Bi-directional pairs and shows the frequency distribution of responses for all ISIs.

Independent stimuli (long ISI): For stimulus 1 and also for stimulus 2, responses at ISI ≥1.4 s, show a main peak at 160 ms, small peaks at 300 and 450 ms and low tail above around 500 ms for the size-weighted integrated delay (Figure 10, last three rows).

Interfering stimuli (short ISI): As ISI decreases the latency of the peak response to stim1 and stim 2 remains unchanged for all ISIs. However, as ISI decreases, responses to stimulus 2 show a reduction in height of the main peak and increase of cases in the tail of size integrated RT2 (Figure 10 all rows). For bidirectional pairs only, statistical analysis (linear mixed effect) confirms a significant effect of ISI on mean size integrated RT2 (F7, 38.9 = 5.3, *p* = 0.0003).

Figure 11 shows the distribution of size-integrated delays at each ISI, separated into bidirectional and unidirectional pairs. Responses to stim1: As ISI decreases below 0.55 s, the latency of tail of the distribution reduces (Figure 11).

Responses to stim2: As ISI decreases ISI (≤0.8s bidirectional, ≤0.25 s unidirectional), the latency of the tail of the distribution increases. The 50th, 75th and 95th percentiles show an increased delay of approximately 100 ms as ISI decreases from long (independent) to short (interfering) for bidirectional stimuli (Figure 11). For unidirectional stimuli the increase is smaller (∼50 ms) and confined to lower ISI (≤0.25 s unidirectional). However, the mode integrated delay changes little if at all at short ISI.

## Discussion

We used impulsive stimuli to investigate balance for a task where participants use myoelectric signals from their leg muscles to control sway of their whole body (Figure 1).

### Principal findings

This study establishes new evidence regarding non-linear processes in balance control at short inter-stimulus-intervals (ISI).

1) While onset of balance response occurs sharply at reflex latency, the complete response includes visible, subsequent components beyond the long-latency reflex (Figure 4).

2) The closed loop control signal u shows systematic non-linearity at short ISI ≤0.55 s (Figure 5).

3) A non-parametric model allowing two non-linear processes, (one fixed delay one variable delay) reproduces the non-linear closed-loop response to double stimuli (Figure 6).

4) The main strength of the two-process response comes from variable delay processes beyond the long-latency reflex (Figure 8). Sensitivity to direction of stimulus2 relative to stimulus1 arises at variable delay latencies beyond the long-latency reflex (Figure 8D).

5) The non-linear balance response shows refractoriness at ISI ≤0.55 s revealed by:

1) insensitivity of an ongoing response defined by stimulus1 to the direction of stimulus2 (Figures 3A, 8A,B,D).
2) non-linear excitation by the arrival of stimulus2, of response to stimulus1 in the direction for stimulus1, (e.g., ISI 0.15 s, Figures 3A, 8A. These triggered reactions are refractory to the direction but not the occurrence of stimulus2.
3) non-linear suppression of response to stimulus2 at short ISI when stimulus 2 is in the opposite direction to stimulus1 (Figures 9E,G,H).

### Validity of the modelling strategy

This study models the relationship between independent disturbance d and experimental control signal u (Figure 1). In whole body balance, the control signal is part of a feedback system in which maintenance of balance requires rejection of the disturbance. Thus the experimental control signal includes a component related to the disturbance and a larger remnant (van der Kooij and Peterka, 2011; Loram et al., 2022). It is believed widely that the balance control system is linear (van der Kooij and de Vlugt, 2007; Kiemel et al., 2011; van der Kooij and Peterka, 2011; Safavynia and Ting, 2013b; Crevecoeur and Kurtzer, 2018; Kurtzer, 2019). To test the basic assumption of linearity, it is sufficient to treat the balance system as a “black box” and it is not necessary to model processes within the system such as the relationship between body position y and control signal u (Figure 1) (Engelhart et al., 2015). The assumption of linearity is tested by the presence or absence of significant, systematic difference between model simulation and experimental signal at all ISI. Note that the ARX model is exactly equivalent to a state-space model (Phan and Lngman, 1996) (Supplementary Figure S1). Any linear state-space system representing the states of a plant and state feedback is transformable into an ARX model of appropriate order. Furthermore, all linear systems follow the principle of superposition, namely that the net response caused by two or more stimuli is the sum of the responses that would have been caused by each stimulus individually. Any deviation from this behavior at low ISI is evidence of non-linearity.

Deviation from linearity: Using AIC criteria to select model order, the high order linear-time-invariant response to the independent stimuli is defined by model M1. Non-linearity at short ISI≤0.55 s is shown unambiguously by systematic difference between the experimental control signal and the LTI model M1 (Figure 5). That systematic difference and its timing is verified using well established methods for statistical analysis of the time-varying F-statistic (1-d SPM, (Pataky, 2012)). A clear example of refractoriness at ISI = 0.15 s is shown in representative data which shows a response excited by stimulus2 but in the direction defined 300 ms earlier by stimulus1 (Figure 4A).

Investigation of non-linearity: Models M2 and M3 are descriptive and capture the experimental control signal with minimal assumptions. However, models M2 and M3 are constrained in that their model structure represents prior empirical evidence demonstrating the existence of reflex and voluntary processes (Rothwell et al., 1980; Marsden et al., 1981; Pruszynski and Scott, 2012). These models hypothesize one process at fixed latency where response amplitude can vary through time and one later process where response amplitude and delay can both vary through time. The variable amplitude fixed delay process represents known ability of the nervous system to vary gain of the long-latency reflex (Pruszynski and Scott, 2012). However, while it known that gain of the long-latency reflex can vary with intention, task and environmental constraints, variation with ISI has not previously been demonstrated. The variable amplitude, variable delay process represents voluntary components which are known to span a wide range of latencies (Marsden et al., 1981; Marsden et al., 1983; Brooks, 1986; Pruszynski and Scott, 2012).

It is expected that increasing the number of parameters will increase the variance percentage fit of the model. However, validity is not determined by an increase in fit to experiment between models M1 and M3. Validity and discrimination between models lies in ability to reproduce the experimental control signal u at all ISI without systematic error: this criterion ruled out models M1 and M2.

Model, M3 represents the dynamics of the closed-loop balance system by a continuous, linear-time-invariant (LTI) ARX model. The denominator coefficients of the ARX model determine the intrinsic system response and the numerator coefficients filter or color any input provided to the system. Non-linear variation in amplitude of the fixed delay response is modelled by optimizing discretely the amplitude of each stimulus applied at the fixed delay. Non-linear variation in amplitude and delay of the second process is modelled by optimizing the amplitude and timing of a second stimulus applied to the LTI system after each fixed delay process. Dynamics of the closed loop response defined by the ARX model coefficients are constant for the whole trial, and the analysis is non-parametric (i.e., many parameters) because in addition to the ARX coefficients it uses 193 parameters (M3) to describe the sizes and delays at reflex latency and variable delay. Optimization of stimulus parameters proceeded sequentially stimulus by stimulus from the last to the first stimulus.

This model structure M3 represents the generalized idea that a real sensory stimulus is processed and provides input to a continuous motor system by two pathways: the first pathway is the shortest possible transcortical input to the motor system, the second pathway follows a longer route allowing variable time before sensory input is resolved into a stimulus to the motor system. This non-parametric analysis including model M3, is agnostic regarding any parametric model of balance and specifically is agnostic as to whether closed loop control of balance is continuous or intermittent. This non-parametric analysis describes the non-linearity which any valid parametric control model should be able to explain.

Statistical analysis of cumulative rather than instantaneous responses is valid when latency is variable because it tests strength at each latency of the response arriving from all preceding latencies. SPM analysis of time-varying significance (Pataky, 2012) shows the temporal onset of significant difference and shows how the effect of refractoriness begins at reflex latency but increases to show a strongest effect within the voluntary period (Figure 8G).

### The physiological validity of refractoriness

Given the 48 ms delay within the Trigno-EMG system, an observed delay of 150–160 ms from onset of stimulus (Figures 4, 5, row1) represents a latency for muscle activity of 102–112 ms. This latency is consistent with the known muscle activation delay (90–120 ms) of all muscles in response to whole-body perturbations (Safavynia and Ting, 2013a). This response is mediated by the fastest possible transcortical reflex integrating multimodal sensation with task level goals (Crevecoeur and Kurtzer, 2018) and is referred to as long-latency reflex (Safavynia and Ting, 2013b). Discrete components following the initial response are observable as discontinuities in representative data (Figure 4) at delays ranging beyond 170 ms up to 1,000 ms (Figure 7, row3): these physiological delays >120 ms for the lower limb are referred to as voluntary responses (Crevecoeur and Kurtzer, 2018).

Refractoriness demonstrated in manual control, is known to occur at short ISIs when processing of a current stimulus requires completion of a serial process initiated previously (Loram et al., 2014). Serial processes are associated with events related to signals crossing thresholds. Neural processing is inherently threshold based with examples ranging from excitation of action potentials in individual neurons to motor decision of one action (Cisek and Kalaska, 2005). Serial processes that could explain refractoriness in balance include 1) accumulation of prediction error until it crosses a threshold stimulating intermittent sampling of sensory input and abrupt change in continuous motor output (Gawthrop et al., 2011), and 2) a possible temporal overhead for selection, preparation and action of a different muscle group (Michalski et al., 2020).

### Discussion of the findings considering other published work

This study complements literature which portrays the long-latency reflex as the main contributor to human balance (Pruszynski and Scott, 2012; Safavynia and Ting, 2013b; Crevecoeur and Kurtzer, 2018; Kurtzer, 2019). We found the long-latency reflex is just the first of a sequence of events which also includes later voluntary components, the sum of which corrects for disturbances (Figures 4, 5). Our observations concur with work in the upper limb showing the unreliability and partial efficacy of the long-latency reflex (Marsden et al., 1981) and showing how the voluntary component compensates for the limited strength, pulsatile nature and variability of the long-latency reflex (Marsden et al., 1983). While the directional action of long-latency reflexes is appropriate for current stimulus direction, we found the long-latency reflex was not sensitive to direction of a stimulus in relation to direction of a previous stimulus (Figure 8F): this insensitivity to historical context appears to be a new result published here for the first time. We found the long-latency reflex showed no evidence of altered delays associated with ISI (Figures 9E,F) which concurs with a review by (Crevecoeur and Kurtzer, 2018) and with a study by (Kurtzer, 2019). However, our results suggest that sensitivity of response amplitude to ISI starts at long latency (Figures 9E–G). Sensitivity of reflex amplitude to ISI in the upper limb was not investigated by (Kurtzer, 2019).

Components within the voluntary timescale provided the largest contribution to response (Figure 7C), reduced coefficient of variation (Figure 7D), provided sensitivity to context namely direction of a stimulus in relation to a previous stimulus (Figure 7F) and showed evidence of suppression of response related to ISI (Figures 7E, 8, 9). This suppression and delayed development of response related to ISI is a hallmark of refractoriness and evidence of serial, threshold related processing. While extensive literature demonstrates refractoriness in manual control (Levy et al., 2006; van de Kamp et al., 2013; Loram et al., 2014), these results show for the first time evidence of refractoriness in human balance. The timescale of refractoriness in balance at ISI≤0.55s is similar to manual control (van de Kamp et al., 2013). This similar timescale suggests a common central process may be responsible for regulating both balance and manual control.

Central structures such as cerebellar-basal ganglia loops modulate long-latency reflexes (Nashner, 1976) and select voluntary responses following evaluation of environmental context (Cisek and Kalaska, 2010; Frank, 2011; Caligiore et al., 2017). The transcortical long-latency reflex provides the fastest possible pathway for integrating multimodal sensation into an integrated whole-body response (Crevecoeur and Kurtzer, 2018). The long-latency reflex cannot be delayed, it can only be suppressed or amplified (Brooks, 1986). Later responses within the voluntary timescale can be delayed (Tatton and Lee, 1975; Brooks, 1986). Motor expression of a central, serial process sensitive to ISI would be distributed and can include 1) suppression of the long-latency reflex and 2) increased delay and suppression of variable delay processes (Loram et al., 2014).

### Conceptual models of balance control

Continuous sensorimotor feedback of task relevant error with a delay corresponding to the long-latency reflex provides a widely supported concept of balance control (Kiemel et al., 2011; Safavynia and Ting, 2013a; Crevecoeur and Kurtzer, 2018). However, this concept ignores the temporally structured, discontinuous character of unaveraged, representative motor output (Figure 4). Threshold related intermittent control concurs with basic principles of neural processing and offers a more ecological-physiological concept of balance control that recognizes a higher bandwidth of task relevant decision making (Morasso et al., 2014; Loram et al., 2015b; Morasso et al., 2020). The systematic non-linearity and refractoriness related to short ISI (Figures 5, 7–9) provides support for intermittent control of balance. Consistent with manual tracking of visual targets (van de Kamp et al., 2013), the effect of ISI was stronger for bidirectional than unidirectional stimulus pairs (Figure 10). This effect of ISI might be related to muscle group selection. For unidirectional pairs, the muscles used to respond to second stimulus are the same as for first stimulus. The larger ISI effect on bidirectional responses might reflect additional time to stop one muscle group and select a new one (Horstmann, 2003; Michalski et al., 2020). Alternatively, refractoriness is known to disappear when stimuli are predictable [Figure 2, (Navas and Stark, 1968),]. If responses are triggered by prediction error exceeding a threshold, then when the second stimulus is in the opposite direction to first (bidirectional), the second stimulus arrival might cancel the expected perturbative effect of the first stimulus and therefore the prediction error threshold is exceeded later. Differences between uni and bidirectional pairs increases support for intermittent control.

### Significance of the work

This study provides new evidence of refractoriness in human balance and re-balances literature which portrays the long-latency reflex as the main contributor to balance control. These results show that voluntary variable delay processes are central to the regulation of balance. These variable delay processes depend upon central function including cerebellar-basal ganglia loops. This new knowledge is relevant to understand how neurological conditions including Parkinson’s disease, dystonia and cerebellar ataxia impair balance.

## Data Availability

The raw data supporting the conclusions of this article will be made available by the authors, without undue reservation.
