# Supplementary material for: What is the contribution of voluntary and reflex processes to sensorimotor control of balance?
Source: Front Bioeng Biotechnol. 2022 Sep 29;10:973716. doi: 10.3389/fbioe.2022.973716 (PMC9557221; doi:10.3389/fbioe.2022.973716)
Supplement: Supplementary file 1 [file Table1.docx]

***Supplementary Appendix 1. Control signal.*** *The control signal (integrated EMG) applied as net torque to the WBM was generated by a myoelectric interface sampling plantar flexion and dorsi-flexion action of both calf and tibialis anterior muscles. The interface was implemented by a multichannel surface electromyograph (sEMG) (Trigno, Delsys) with a sample frequency of 2 kHz and Ag/AgCl electrodes were used to measure the electrical activity of leg muscles Tibialis Anterior (TA) and calf muscles (intersection of Gastrocnemius Medialis and Soleus (G) of both right and left leg. Electrodes placement was accomplished according to SENIAMs (Surface Electromyography for the Non-Invasive Assessment of Muscles) recommendations [59]. Once the electrodes were in place, the electrical activity in all muscles was recorded while muscles were at rest in order to remove noise due to spontaneous electrical activity, not corresponding to muscle work. Those dead-zone values were measured at the beginning of each experimental session. Throughout the task, sEMG signals were processed in real-time through a high pass filter (cut-toff 10 Hz, then rectified and then low-pass filtered (second order transfer function with two time constants of 100 ms). The specific control signal was generated by the sum of the muscular contributions of the two legs evaluated as the sEMG envelops signals difference between the two antagonist muscles (TA and G).*

***Supplementary Video 1. Illustration of motion and actuation of the apparatus.*** *The apparatus (WBM) is actuated myoelectrically. In this video, sEMG signals from two arm muscles are acquired and used to generate the control signal. This signal drives a second order system (virtual inverted pendulum). The real WBM is actuated by a direct drive linear servotube to rotate around its single axis of rotation. The position of the WBM is controlled to follow the position of the virtual inverted pendulum with a delay of approximately 4 ms. With a longer delay, the position is displayed visually on the screen by the green ball. In this example, for visibility, contraction of deltoid makes the WBM lean forwards; activation of the triceps makes the WBM lean backwards. This video illustrates the process of controlling the WBM using myoelectric signals. Our experimental setup, which mimics postural balance, is shown in S4 Video.*

***Supplementary Video 2. Illustration of experimental setup.*** *The participant who is strapped to the apparatus (WBM) stands upright with their feet on horizontal surface fixed to the ground. The WBM is a single segment board which rotates around a single axis of rotation aligned approximately with the human ankle joints. The WBM is actuated myoelectrically: sEMG signals from Tibialis Anterior and Calf muscles in both legs are acquired and used to generate the control signal representing a combined net ankle torque. The control is myoelectric only and contains no passive component. The WBM provides the participant with haptic feedback, natural visual feedback and natural vestibular feedback of motion of the unstable second order system (virtual inverted pendulum with time constant of an adult human) rotating around the real axis of rotation in line approximately with the participant ankle joints.*

***Supplementary Figure 1. Example disturbance d, experimental control signal u with illustrative simulations using ARX and other models fitted to the data (d, u)****. The high order ARX model (uARX) was fitted using the procedure described for model M1. The state space model (uSS) is a state-space representation of the high order ARX model produced using MATLAB function ‘idss’ to convert the ARX model to state-space form. The parametric non-predictive linear state estimation model with added noise (NPC) was fitted following the procedure given in (Loram et al., 2022). Top Row: Experimental control signal (blue), High Order ARX model (red), statespace form of ARX model (yellow), parametric linear state estimation model (magenta). Middle Row: High Order ARX model (blue), state-space form of ARX model (red). Bottom Row: Disturbance (blue). Message: The ARX polynomial input-output form model is identical to its state-state form.*
