# Supplementary figures and images for: What is the contribution of voluntary and reflex processes to sensorimotor control of balance?
Source: Front Bioeng Biotechnol. 2022 Sep 29;10:973716. doi: 10.3389/fbioe.2022.973716 (PMC9557221; doi:10.3389/fbioe.2022.973716)

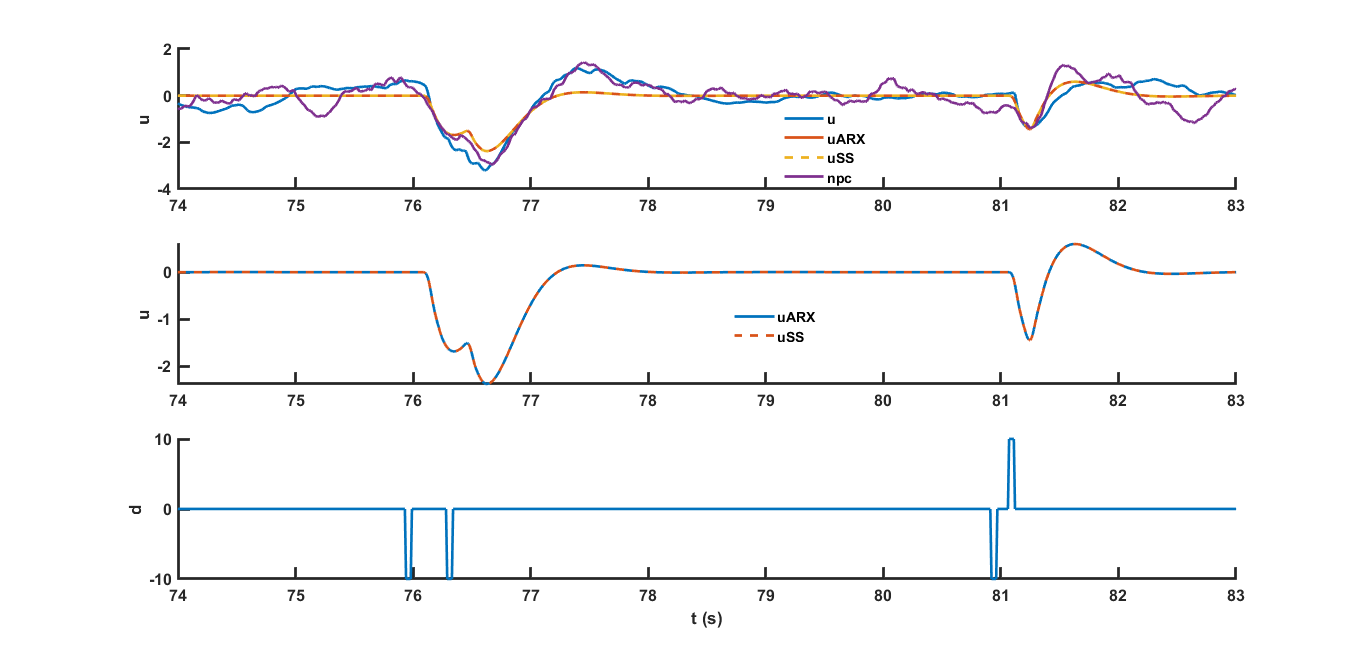

Supplement: Supplementary file 3 [file Image1.PNG]
